# Copper-catalyzed *O*-alkenylation of phosphonates

**DOI:** 10.3762/bjoc.16.56

**Published:** 2020-04-03

**Authors:** Nuria Vázquez-Galiñanes, Mariña Andón-Rodríguez, Patricia Gómez-Roibás, Martín Fañanás-Mastral

**Affiliations:** 1 Centro Singular de Investigación en Química Biolóxica e Materiais Moleculares (CiQUS), Departamento de Química Orgánica, Universidade de Santiago de Compostela, 15782 Santiago de Compostela, Spain

**Keywords:** alkenylation, copper, C(sp^2^)–O bond formation, hypervalent iodine, phosphonates

## Abstract

Copper catalysis allows the direct oxygen alkenylation of dialkyl phosphonates with alkenyl(aryl)iodonium salts with selective transfer of the alkenyl group. This novel methodology proceeds with a wide range of phosphonates under mild conditions and gives straightforward access to valuable enol phosphonates in very good yields.

## Introduction

**Scheme 1:**
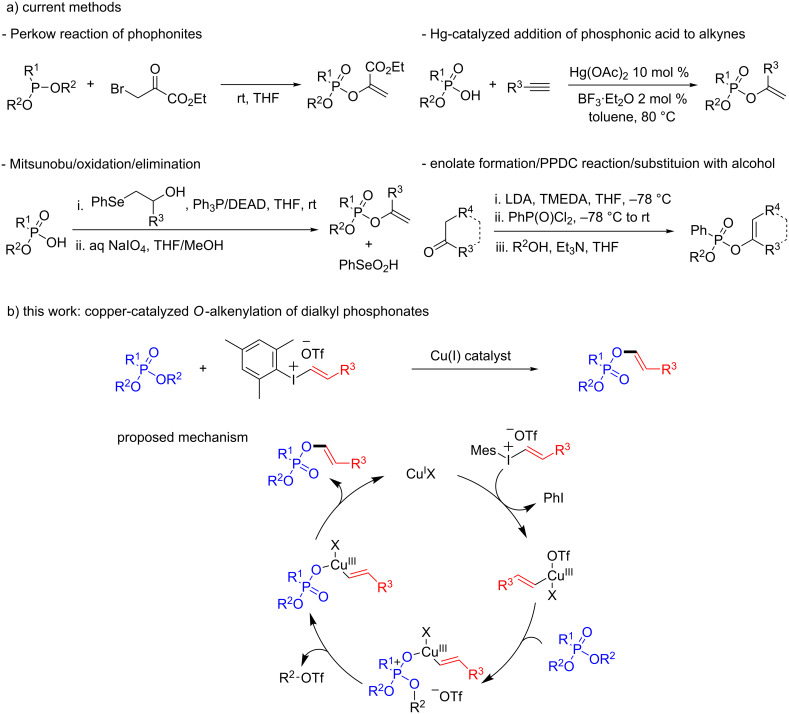
Synthesis of mixed alkyl alkenyl phosphonates.

Organophosphorus compounds represent an important class of products with a wide range of applications in biology, agriculture and synthetic organic chemistry [1–3]. In particular, *O*-alkenyl phosphonate esters (i.e., enol phosphonates) have been described as potent insecticides and show antifungal activity [4]. While several methods are available for the preparation of cyclic enol phosphonates [5–10], the synthesis of the acyclic counterparts has received less attention. Current methodologies for the synthesis of acyclic mixed enol phosphonates include the Perkow-type reaction between phosphonites and α-halocarbonyl compounds [11], the mercury-catalyzed addition of phosphonic acid monoesters to terminal alkynes [12–13] and multistep procedures involving a Mitsunobu reaction between 2-hydroxyalkyl phenyl selenides and phosphonic acid monoesters followed by an oxidation/elimination step [14] or reaction of an enolate with a phosphonic dichloride and subsequent treatment with an alcohol [15] (Scheme 1a). However, these procedures are subject to selectivity problems, involve toxic and hazardous materials or are limited to the restricted availability of the corresponding phosphorus reagents. Therefore, the development of alternative methods for the synthesis of acyclic enol phosphonates is highly desirable.

Diaryliodonium and aryl(alkenyl)iodonium salts, which are air- and moisture-stable, nontoxic and easy to prepare compounds, have become efficient reagents for mild and selective arylation and alkenylation reactions in organic synthesis [16–18]. In particular, the use of these hypervalent iodine reagents in copper catalysis has allowed to perform a wide range of previously unknown synthetic transformations [19–29]. In these reactions, aryl(vinyl)Cu(III) species [30–31] have been proposed as key intermediates to undergo reactions with a variety of nucleophiles. Fañanás-Mastral and Feringa recently reported a catalytic method for the synthesis of mixed alkyl aryl phosphonates based on a copper-catalyzed arylation of phosphonates with diaryliodonium salts [32]. Encouraged by this work, in the context of an electrophilic alkenylation of phosphonates, we reasoned that the action of a copper catalyst on an alkenyl(aryl)iodonium salt [33–34] would generate an alkenyl–copper(III) species which might undergo nucleophilic attack of the Lewis-basic oxygen of a dialkyl phosphonate. The resulting phosphonium-like intermediate would evolve by Arbuzov-type substitution of one of the alkyl groups, and final reductive elimination would form the new C(sp^2^)–O bond, providing an acyclic enol phosphonate with concomitant regeneration of the Cu(I) catalyst (Scheme 1b). Herein we report the successful realization of such a copper-catalyzed oxygen-alkenylation strategy and show that a range of readily available, dialkyl phosphonates and alkenyl(aryl)iodonium salts can be combined to form enol phosphonates in high yield and excellent selectivity.

## Results and Discussion

We started our studies by investigating the reaction between diethyl phosphonate **1a** and styryl(mesityl)iodonium triflate (**2a**, Table 1). We first run the reaction under the conditions reported for the copper-catalyzed O-arylation of phosphonates (CuCl as catalyst, 2,6-di-*tert*-butylpyridine (dtbpy) as additive in dichloromethane at 40 °C) [32]. Under those conditions, enol phosphonate **3a** was the only product of the reaction, although low conversion and yield were observed (Table 1, entry 1). A screening of copper complexes at a higher temperature (50 °C) revealed that CuTC (TC = thiophene-2-carboxylate) is the most efficient catalyst for this transformation (Table 1, entries 2–6). Finally, by using 2 equiv of **2a** full conversion was achieved and enol phosphonate **3a** was isolated in 78% yield with full selectivity towards the monoalkenylation product (Table 1, entry 7). Importantly, no reaction was observed in the absence of copper catalyst (Table 1, entry 8), while the absence of dtbpy led to a minimal conversion (Table 1, entry 9). The structure of the alkenyliodonium salt also plays an important role in the outcome of the reaction since the use of a phenyl group instead of the mesityl ligand caused a dramatic decrease in conversion and reaction yield likely due to a faster decomposition of the salt (Table 1, entry 10).

**Table 1:** Optimization studies^a^.

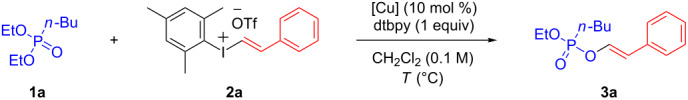					

entry	**2a** (equiv)	[Cu]	*T* (°C)	conv (%)^b^	**3a** (%)^b^

1	1.5	CuCl	40	42	34
2	1.5	CuCl	50	63	53
3	1.5	CuOTf·PhCH_3_	50	32	25
4	1.5	Cu(OTf)_2_	50	65	60
5	1.5	CuI	50	50	50
6	1.5	CuTC	50	75	69
7	2	CuTC	50	full	82 (78)^c^
8	2	–	50	–	–
9^d^	2	CuTC	50	10	5
10^e^	2^e^	CuTC	50	30	15

^a^Reactions run on a 0.2 mmol scale; ^b^Determined by ^1^H NMR using 1,3,5-trimethoxybenzene as internal standard. ^c^Yield of isolated product shown in brackets. ^d^Reaction run in the absence of dtbpy. ^e^Styryl(phenyl)iodonium triflate used instead of **2a**.

**Scheme 2:**
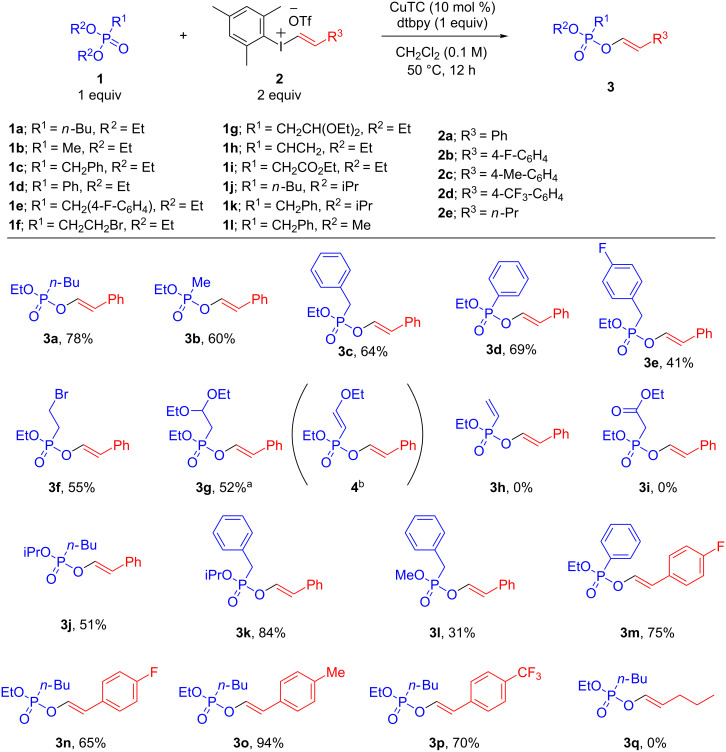
Scope of the copper-catalyzed alkenylation of dialkyl phosphonates. Reactions run on a 0.2 mmol scale. Yields refer to isolated pure products. ^a^Reaction time = 10 h. ^b^When reaction was stirred over 18 h a **3g**:**4** mixture was obtained in a 1:1 ratio.

Having established optimized conditions for the copper-catalyzed O-alkenylation of phosphonates, we set out to investigate the scope of the reaction (Scheme 2). This catalytic transformation proved to be very efficient for several diethyl phosphonates bearing alkyl, benzyl and aryl groups providing in all cases the corresponding enol phosphonates **3a–d** in good yields. Importantly, no double alkenylation product was observed in any case. Benzyl and alkyl diethyl phosphonates bearing halide groups also worked well and led to enol phosphonates **3e** and **3f** in good yields without any traces of side products. An acetal-protected aldehyde could also be used providing enol phosphonate **3g** in 52% yield. In this case, prolonged reaction times led to partial evolution of **3g** into enol ether **4**. This transformation may be explained by an acid-mediated elimination of ethanol likely caused by trace formation of triflic acid via decomposition of ethyl triflate. As a limitation, substrates bearing a vinyl substituent or an enolizable ester group did not give any conversion. This methodology is also applicable to other dialkyl phosphonates as illustrated by the synthesis of enol phosphonates **3j**, **3k** and **3l**. Interestingly, the copper-catalyzed alkenylation of phosphonates followed the same reactivity trend as the one described for the arylation reaction [32] with the diisopropyl phosphonates being more efficient than the dimethyl phosphonate esters. It is also important to remark that, in sharp contrast to the copper-catalyzed reaction between *H*-phosphonates and vinyliodonium salts described by Eustache and co-workers [35], no formation of the P-alkenylation product was observed in any case.

Different alkenyliodonium salts were also used for this transformation. Styryl(mesityl)iodonium salts bearing both electron-donating and electron-withdrawing substituents worked well and allowed access to the corresponding enol phosphonates **3m–p** in very good yields. Importantly, the bulky mesityl ligand allowed the selective transfer of the alkenyl group in all cases. In sharp contrast, no alkenylation product was observed when alkenyliodonium salts bearing aliphatic substituents were used likely due to a faster decomposition of the salt [36–37].

## Conclusion

In summary, we have developed an efficient copper-catalyzed oxygen alkenylation of dialkyl phosphonates with alkenyl(aryl)iodonium salts. The reaction proceeds under mild conditions, with excellent levels of selectivity and affords acyclic enol phosphonates in high yields. We believe that the reaction occurs through the formation of a high valent alkenyl–copper(III) species which gets attacked by the phosphoryl oxygen of the phosphonate.

## Supporting Information

File 1 Experimental procedures and characterization data of enol phosphonates **3**.

## References

[1] McGrath J W, Chin J P, Quinn J P (2013). Nat Rev Microbiol.

[2] Duke S O, Powles S B (2008). Pest Manage Sci.

[3] Quin L D (2000). A Guide to Organophosphorus Chemistry.

[4] Engel R (1992). Handbook of Organophosphorus Chemistry.

[5] Peng A-Y, Ding Y-X (2003). J Am Chem Soc.

[6] Peng A-Y, Ding Y-X (2004). Org Lett.

[7] Unoh Y, Hashimoto Y, Takeda D, Hirano K, Satoh T, Miura M (2013). Org Lett.

[8] Seo J, Park Y, Jeon I, Ryu T, Park S, Lee P H (2013). Org Lett.

[9] Park Y, Seo J, Park S, Yoo E J, Lee P H (2013). Chem – Eur J.

[10] Pérez-Saavedra B, Vázquez-Galiñanes N, Saá C, Fañanás-Mastral M (2017). ACS Catal.

[11] Despax C, Navech J (1990). Tetrahedron Lett.

[12] Peng A, Ding Y (2003). Synthesis.

[13] Wasserman H H, Cohen D (1960). J Am Chem Soc.

[14] Sheng S-R, Sun W-K, Hu M-G, Liu X-L, Wang Q-Y (2007). J Chem Res.

[15] Campbell I B, Guo J, Jones E, Steel P G (2004). Org Biomol Chem.

[16] Zhdankin V V, Stang P J (2008). Chem Rev.

[17] Merritt E A, Olofsson B (2009). Angew Chem, Int Ed.

[18] Aradi K, Tóth B L, Tolnai G L, Novák Z (2016). Synlett.

[19] Fañanás-Mastral M (2017). Synthesis.

[20] Phipps R J, Grimster N P, Gaunt M J (2008). J Am Chem Soc.

[21] Phipps R J, Gaunt M J (2009). Science.

[22] Zhu S, MacMillan D W C (2012). J Am Chem Soc.

[23] Suero M G, Bayle E D, Collins B S L, Gaunt M J (2013). J Am Chem Soc.

[24] Collins B S L, Suero M G, Gaunt M J (2013). Angew Chem, Int Ed.

[25] Xu Z-F, Cai C-X, Liu J-T (2013). Org Lett.

[26] Wang Y, Chen C, Peng J, Li M (2013). Angew Chem, Int Ed.

[27] Cahard E, Male H P J, Tissot M, Gaunt M J (2015). J Am Chem Soc.

[28] Beaud R, Phipps R J, Gaunt M J (2016). J Am Chem Soc.

[29] Teskey C J, Sohel S M A, Bunting D L, Modha S G, Greaney M F (2017). Angew Chem, Int Ed.

[30] Hickman A J, Sanford M S (2012). Nature.

[31] Casitas A, Ribas X (2013). Chem Sci.

[32] Fañanás-Mastral M, Feringa B L (2014). J Am Chem Soc.

[33] Ochiai M, Sumi K, Nagao Y, Fujita E (1985). Tetrahedron Lett.

[34] Okuyama T, Takino T, Sato K, Ochiai M (1998). J Am Chem Soc.

[35] Thielges S, Bisseret P, Eustache J (2005). Org Lett.

[36] Beringer F M, Bodlaender P (1969). J Org Chem.

[37] Lockhart T P (1983). J Am Chem Soc.

