# Supplementary material for: Copper-catalyzed O-alkenylation of phosphonates
Source: Beilstein J Org Chem. 2020 Apr 3;16:611–5. doi: 10.3762/bjoc.16.56 (PMC7136550; doi:10.3762/bjoc.16.56)
Supplement: File 1 — Experimental procedures and characterization data of enol phosphonates 3. [file Beilstein_J_Org_Chem-16-611-s001.pdf]

BEILSTEIN JOURNAL OF ORGANIC CHEMISTRY

Supporting Information

for

Copper-catalyzed *O*-alkenylation of phosphonates

Nuria Vázquez-Galiñanes, Mariña Andón-Rodríguez, Patricia Gómez-Roibás and Martín Fañanás-Mastral

Beilstein J. Org. Chem. **2020,** *16,* 611–615. doi:10.3762/bjoc.16.56

Experimental procedures and characterization data of enol phosphonates 3

License and Terms: This is a supporting information file under the terms of the Creative Commons Attribution License (http://creativecommons.org/licenses/by/4.0). Please note that the reuse, redistribution and reproduction in particular requires that the authors and source are credited.
The license is subject to the *Beilstein Journal of Organic Chemistry* terms and conditions: (https://www.beilstein-journals.org/bjoc)

Table of contents

**1.**	**General methods**	**S2**
**2.**	**List of starting materials**	**S3**
**3.**	**General procedure for the copper-catalyzed oxygen-alkenylation of dialkyl phosphonates**	**S4**
**4.**	**Compound characterization**	**S4**
**5.**	**^{1}H NMR and ^{13}C NMR spectra**	**S9**

1. General methods

• All reactions were performed under argon atmosphere using oven dried glassware and using a sealed tube. Solvents were dried using an MBraun SPS 800 system. All chemicals and copper complexes were purchased from Acros Organics Ltd., Aldrich Chemical Co. Ltd., Alfa Aesar, Apollo, Strem Chemicals Inc., Fluorochem Ltd. or TCI Europe N.V. chemical companies and used without further purification, unless otherwise noted.

• Analytical thin layer chromatography was carried out on silica-coated aluminium plates (silica gel 60 F254 Merck) and components were visualized by UV light, I2 and $KMnO_4$ staining. Flash column chromatography was performed on silica gel 60 (Merck, 230–400 mesh) without previous deactivation, unless otherwise stated.

• High resolution mass spectrometry was carried out on a Bruker microTOF spectrometer using APCI.

• ^{1}H and ^{13}C NMR experiments were carried out using a Varian Inova 500 MHz or a Varian Mercury 300MHz NMR spectrometers. Chemical shift values are reported in ppm with the solvent resonance as the internal standard ($CHCl_3$: δ 7.26 for ^{1}H, δ 77.16 for ^{13}C). Coupling constants (*J)* are given in Hertz (Hz). Multiplicities are reported as follows: s = singlet, d = doublet, t = triplet, q = quartet, p = pentet, m = multiplet or as a combination of them.

2. List of starting materials

- Phosphonates **1a**, **1b**, **1d**-**1j** were obtained from commercial sources. **1c, 1k, 1l** were prepared according to described procedures.[1]

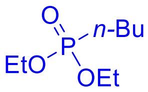

1a

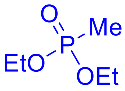

1b

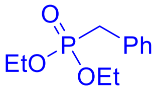

1c

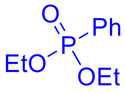

1d

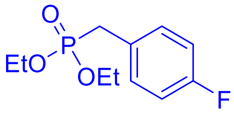

1e

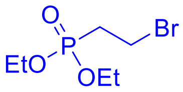

1f

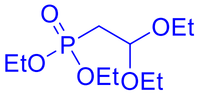

1g

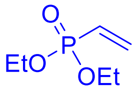

1h

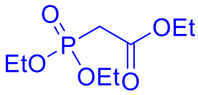

1i

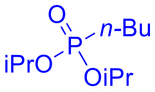

1j

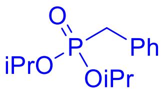

1k

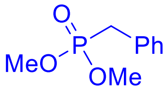

1l

- Alkenyl(mesityl)iodonium salts **2** were prepared according to described procedures.[2,3]

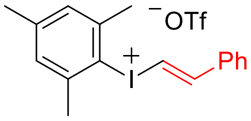

2a

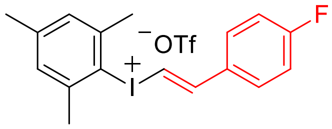

2b

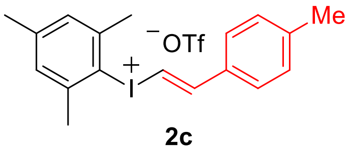

2c

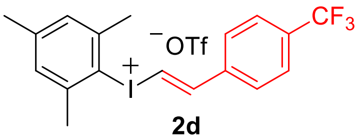

2d

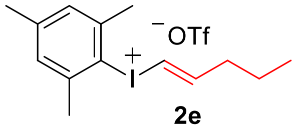

2e

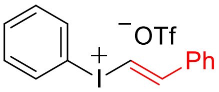

2f

---

[1] Huang, T.; Chen, T.; Han, L., *J. Org. Chem.* **2018**, *83*, 2959–2965.
[2] Suero, M. G.; Bayle, E. D.; Collins, B. S. L.; Gaunt, M. J. *J. Am. Chem. Soc.* **2013**, *135*, 5332–5335.
[3] Ren, W.; Xiang, H.; Peng, C.; Musha, Z.; Chen, J.; Huang, R.; Hu, Y. *RCS Adv*. **2018**, *8*, 5542–5549.

3. General procedure for the copper-catalyzed oxygen-alkenylation of dialkyl phosphonates

CuTC (0.02 mmol, 0.10 equiv), the corresponding alkenyl(aryl)iodonium salt **2** (0.6 mmol, 2 equiv) and a stirring bar were charged in a sealed tube. A solution of phosphonate **1** (0.2 mmol, 1 equiv) in dry dichloromethane (2 ml) and 2,6-di-*tert*-butylpyridine (0.22 mmol, 1 equiv) was added and the resulting mixture was stirred at 50 °C under an argon atmosphere for 16 h. After this time, the reaction was quenched by addition of saturated aqueous solution of NH_4Cl (10 ml) and extracted with CH_2Cl (2 × 10 ml). The organic layer was dried over Na_2SO_4, filtered and solvent was removed under vacuum. The final product was purified by column chromatography on silica gel using *n*-hexane/AcOEt (1:1) as eluent.

4. Compound characterization

(*E*)-Ethyl styryl butylphosphonate (3a)

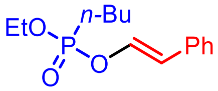

Obtained from **1a** and **2a** following the general procedure as a yellow oil after column chromatography following the general procedure in 78% yield.

^{1}H NMR (300 MHz, $CDCl_3$) δ 7.3 – 7.17 (m, 6H), 6.34 (dd, *J* = 12.4, 1.5 Hz, 1H), 4.29 – 4.08 (m, 2H), 1.93 – 1.77 (m, 2H), 1.73 – 1.53 (m, 2H), 1.49 – 1.38 (m, 2H), 1.34 (t, *J* = 7.1 Hz, 3H), 0.92 (t, *J* = 7.4 Hz, 3H). **^{13}C NMR** (126 MHz, $CDCl_3$) δ 137.1 (d, *J* = 6.0 Hz, CH), 134.2 (C), 128.7 (2 x CH), 127.2 (CH), 126.0 (2 x CH), 116.7 (d, *J* = 9.5 Hz, CH), 62.0 (d, *J* = 7.1 Hz, CH_2), 25.3 (d, *J* = 140.2 Hz, CH_2), 24.1 (d, *J* = 5.3 Hz, CH_2), 23.6 (d, *J* = 17.5 Hz, CH_2), 16.4 (d, *J* = 6.0 Hz, CH_3), 13.5 (CH_3). **^{31}P NMR** (202 MHz, $CDCl_3$) δ32.65. **HRMS** (APCI) Calc. for $C_{14}H_{22}O_3P$ [M+H$^+$]: 269.1300, found 269.1301.

(*E*)-Ethyl styryl methylphosphonate (3b)

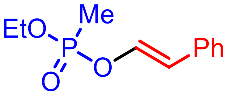

Obtained from **1b** and **2a** following the general procedure as a yellow oil after column chromatography following the general procedure in 60% yield.

^{1}H NMR (500 MHz, $CDCl_3$) δ 7.33 – 7.29 (m, 4H), 7.26 – 7.21 (m, 2H), 6.40 (dd, *J* = 12.4, 1.5 Hz, 1H), 4.29 – 4.15 (m, 2H), 1.62 (d, *J* = 17.8 Hz, 3H), 1.39 (t, *J* = 7.1 Hz, 3H). **^{13}C NMR** (126 MHz, $CDCl_3$) δ 136.7 (d, *J* = 6.1 Hz, CH), 134.0 (C), 128.5 (2 x CH), 127.3 (CH), 126.0 (2 x CH), 117.3 (d, *J* = 9.6 Hz, CH), 62.2 (d, *J* = 6.7 Hz, CH_2), 16.3 (d, *J* = 6.3 Hz, CH_3), 11.2 (d, *J* = 145.3 Hz, CH_3). **^{31}P NMR** (202 MHz, $CDCl_3$) δ 30.52. **HRMS** (APCI) Calc. for $C_{11}H_{16}O_3P$ [M+H$^+$]: 227.0832, found 227.0832.

(*E*)-Ethyl styryl benzylphosphonate (3c)

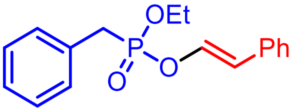

Obtained from **1c** and **2a** following the general procedure as a yellow oil after column chromatography following the general procedure in 64% yield.

^{1}H NMR (300 MHz, $CDCl_3$) δ 7.36 – 7.18 (m, 10H), 7.09 (dd, *J* = 12.3, 6.4 Hz, 1H), 6.30 (dd, *J* = 12.1, 1.3 Hz, 1H), 4.20 – 4.06 (m, 2H), 3.28 (d, *J* = 21.8 Hz, 2H), 1.28 (t, *J* = 7.0 Hz, 3H). **^{13}C NMR** (126 MHz, $CDCl_3$) δ 137.0 (d, *J* = 6.5 Hz, CH), 134.0 (C),130.4 (d, *J* = 9.5 Hz, C) (129.9 (d, *J* = 6.7 Hz, CH), 128.7 (d, *J* = 3.2 Hz, 2 x CH), 128.8 (2 x CH), 127.3 (2 x CH), 127.2 (d, *J* = 3.8 Hz, CH), 126.0 (2 x CH), 117.1 (d, *J* = 9.5 Hz, CH), 62.94 (d, *J* = 7.3 Hz, CH_2), 33.6 (d, *J* = 138.0 Hz, CH_2), 16.3 (d, *J* = 5.8 Hz, CH_3). **HRMS** (APCI) Calc. for $C_{17}H_{20}O_3P$ [M+H$^+$]: 303.1145, found 303.1145.

(*E*)-Ethyl styryl phenylphosphonate (3d)

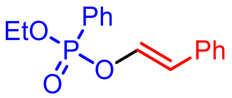

Obtained from **1d** and **2a** following the general procedure as a yellow oil after column chromatography following the general procedure in 69% yield.

^{1}**H NMR** (500 MHz,CDCl$_3$) δ 7.89 (ddd, *J* = 13.7, 8.3, 1.5 Hz, 2H), 7.64 – 7.59 (tq, *J* = 7.4, 1.4 Hz, 1H), 7.55 – 7.50 (m, 2H), 7.32 – 7.28 (m, 4H), 7.27 – 7.20 (m, 2H), 6.41 (dd, *J* = 12.3, 1.2 Hz, 1H), 4.32 – 4.24 (m, 2H), 1.42 (t, *J* = 7.1 Hz, 3H). ^{13}**C NMR** (126 MHz, CDCl$_3$) δ 136.8 (d, *J* = 5.3 Hz, CH), 134.0 (C), 133.0 (d, *J* = 3.1 Hz, CH), 131.8 (d, *J* = 10.2 Hz, 2 x CH), 128.7 (2 x CH), 128.7 (d, *J* = 15.4 Hz, 2 x CH), 127.3 (CH), 127.1 (d, *J* = 191.3 Hz, C), 126.0 (2 x CH), 117.6 (d, *J* = 9.9 Hz, CH), 62.9 (d, *J* = 6.0 Hz, CH$_2$), 16.4 (d, *J* = 6.4 Hz, CH$_3$). ^{31}**P NMR** (202 MHz, CDCl$_3$) δ 18.19. **HRMS** (APCI) Calc. for $C_{16}H_{18}O_3P$ [M+H$^+$]: 289.0990, found 289.0988.

(*E*)-Ethyl styryl 4-fluorobenzylphosphonate (3e)

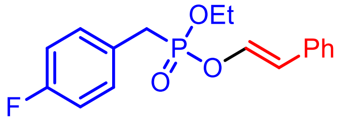

Obtained from **1e**, **2a** following the general procedure as a yellow oil after column chromatography following the general procedure in 41% yield.

^{1}**H NMR** (500 MHz, CDCl$_3$) δ 7.33 – 7.28 (m, 4H), 7.27 – 7.23 (m, 3H), 7.12 (dd, *J* = 12.3, 6.4 Hz, 1H), 7.04 (t, *J* = 8.5 Hz, 2H), 6.33 (dd, *J* = 12.3, 1.4 Hz, 1H), 4.20 –4.12 (m, 2H), 3.27 (d, *J* = 21.3 Hz, 2H), 1.31 (t, *J* = 6.9 Hz, 3H). ^{13}**C NMR** (126 MHz, CDCl$_3$) δ 162.1 (dd, *J* = 246.0, 4.1 Hz, C), 136.9 (d, *J* = 6.4 Hz, 2 x CH), 133.9 (C), 131.4 (dd, *J* = 8.0, 6.8 Hz, 2 x CH), 128.7 (2 x CH), 127.3 (2 x CH), 126.3 (dd, *J* = 9.4, 3.3 Hz, C), 126.0 (2 x CH), 117.2 (d, *J* = 9.6 Hz, CH), 115.6 (dd, *J* = 21.6, 3.1 Hz, CH), 62.9 (d, *J* = 7.3 Hz, CH$_2$), 32.7 (d, *J* = 139.2 Hz, CH$_2$), 16.3 (d, *J* = 5.9 Hz, CH$_3$). ^{31}**P NMR** (202 MHz, CDCl$_3$) δ 24.49. ^{19}**F NMR** (282 MHz, CDCl$_3$) δ -115.45. **HRMS (APCI)** Calc. for $C_{17}H_{19}FO_3P$ [M+H$^+$]: 321.1050 found 321.1048.

(*E*)-Ethyl styryl (2-bromoethyl)phosphonate (3f)

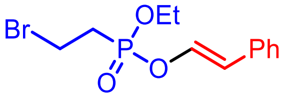

Obtained from **1f** and **2a** as a yellow oil after column chromatography following the general procedure in 55% yield.

^{1}**H NMR** (500 MHz, CDCl$_3$) δ 7.33 – 7.26 (m, 4H), 7.22 (dd, *J* = 12.3, 6.6 Hz, 1H),6.42 (dd, *J* = 12.4, 1.5 Hz, 1H), 4.32 – 4.17 (m, 2H), 3.65 – 3.56 (m, 2H), 2.60 – 2.49 (m, 2H), 1.39 (t, *J* = 7.08 Hz, 3H). ^{13}**C NMR** (126 MHz, CDCl$_3$) δ 136.4 (d, *J* = 6.2 Hz, CH), 133.7 (C), 128.8 (2 x CH), 127.5 (CH), 126.1 (2 x CH), 117.8 (d, *J* = 9.7 Hz, CH), 62.8 (d, *J* = 7.1 Hz, CH$_2$), 30.5 (d, *J* =135.3 Hz, CH$_2$), 22.9 (CH$_2$), 16.4 (d, *J* = 6.0 Hz, CH$_3$). ^{31}**P NMR** (202 MHz, CDCl$_3$) δ 25.35. **HRMS** (APCI) Calc. for $C_{12}H_{17}BrO_3P$ [M+H$^+$]: 319.0095, found 319.0093.

(*E*)-Ethyl styryl (2,2-diethoxyethyl)phosphonate (3g)

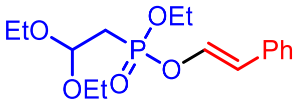

3g

Obtained from **1g** and **2a** as a yellow oil after column chromatography following the general procedure in 52% yield. Reaction time: 10 h.*

^{1}**H NMR** (500 MHz, CDCl$_3$) δ 7.34 – 7.28 (m, 4H), 7.26 – 7.21 (m, 2H), 6.37 (dd, *J* = 12.4, 1.4 Hz, 1H), 4.98 (q, *J* = 5.7 Hz, 1H), 4.27 – 4.17 (m, 2H), 3.72 – 3.67 (m, 2H), 3.62 – 3.55 (m, 2H), 2.36 (dd, *J* = 18.8, 5.8 Hz, 2H), 1.39 (t, 7.1 Hz, 3H), 1.24 (t, *J* = 7.1 Hz, 3H), 1.21 (t, *J* = 7.1 Hz, 3H). ^{13}**C NMR** (75 MHz, CDCl$_3$) δ 137.2 (d, *J* = 13.5 Hz, CH), 134.3 (C), 128.9 (2 x CH), 127.5 (CH), 126.3 (2 x CH), 117.2 (d, *J* = 8.6 Hz, CH), 98.2 (d, *J* = 19.9 Hz, CH), 62.63 (d, *J* = 6.4 Hz, CH$_2$), 62.0 (2 x CH$_2$), 32.0 (d, *J* = 140.4 Hz, CH$_2$), 16.6 (d, *J* = 6.9 Hz, CH$_3$), 15.4 (CH$_3$). ^{31}**P**

NMR (202 MHz, $CDCl_3$) δ 25.28. **HRMS (APCI)** Calc. for $C_{16}H_{26}O_5P$ [M+H$^+$]: 329.1512 found 329.1515.

*Note: when reaction was stirred over 18 h a 1:1 mixture of **3g** and **4** was obtained.

Ethyl (*E*)-styryl ((*E*)-2-ethoxyvinyl)phosphonate (4)

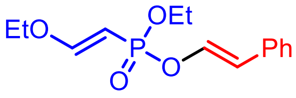

4

^{1}H NMR (500 MHz, $CDCl_3$) δ 7.34 – 7.28 (m, 6H), 7.18 (dd, *J* = 12.4, 6.9 Hz, 1H), 6.37 (dd, *J* = 12.4, 1.4 Hz, 1H), 4.80 (dd, *J* = 13.6, 10.5 Hz, 1H), 4.27 – 4.17 (m, 2H), 3.94 (q, *J* = 7.0 Hz, 2H), 1.42 – 1.34 (m, 6H). **^{13}C NMR** (126 MHz, $CDCl_3$) δ 164.2 (d, *J* = 22.2 Hz, CH), 136.9 (d, *J* = 5.9 Hz, CH), 134.1 (C), 128.7 (2 x CH), 127.2 (CH), 125.9 (2 x CH), 116.9 (d, *J* = 9.9 Hz, CH), 87.0 (d, *J* = 205.2 Hz, CH), 66.5 (CH_2), 62.3 (d, *J* = 6.8 Hz, CH_2), 16.3 (d, *J* = 4.6 Hz, CH_3), 14.3 (CH_3). **^{31}P NMR** (202 MHz, $CDCl_3$) δ 21.43. **HRMS (APCI)** Calc. for $C_{14}H_{20}O_4P$ [M+H$^+$]: 283.1094 found 283.1095.

(*E*)-Isopropyl styryl butylphosphonate (3j)

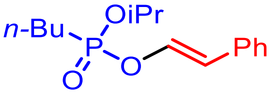

Obtained from **1j** and **2a** as a yellow oil after column chromatography following the general procedure in 51% yield.

^{1}H NMR (300 MHz, $CDCl_3$) δ 7.28 – 7.18 (m, 6H), 6.33 (dd, *J* = 12.4, 1.4 Hz, 1H), 4.86 – 4.75 (m, 1H), 1.90 – 1.77 (m, 2H), 1.71 – 1.55 (m, 2H), 1.42 (sext, *J* = 7.4 Hz, 2H), 1.35 (dd, *J* = 6.2, 3.8 Hz, 6H), 0.92 (t, *J* = 7.3 Hz, 3H). **^{13}C NMR** (126 MHz, $CDCl_3$) δ 137.4 (d, *J* = 6.0 Hz, CH), 134.5 (C), 128.9 (2 x CH), 127.4 (CH), 126.2 (2 x CH), 116.9 (d, *J* = 9.5 Hz, CH), 71.3 (d, *J* = 7.2 Hz, CH), 26.0 (d, *J* = 141.0 Hz, CH_2), 24.4 (d, *J* = 5.5 Hz, CH_2), 24.3 (d, *J* = 5.0 Hz, CH_3), 24.2 (d, *J* = 4.8 Hz, CH_3), 23.9 (d, *J* = 17.7 Hz, CH_2), 13.81 (d, *J* = 1.3 Hz, CH_3). **^{31}P NMR** (202 MHz, $CDCl_3$) δ31.70. **HRMS** (APCI) Calc. for $C_{15}H_{24}O_3P$ [M+H$^+$]: 283.1458, found 283.1460.

(*E*)-Isopropyl styryl benzylphosphonate (3k)

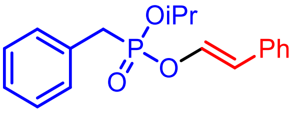

Obtained from **1k** and **2a** as a yellow oil after column chromatography following the general procedure in 84% yield.

^{1}H NMR (500 MHz, $CDCl_3$) δ 7.36 – 7.34 (m, 4H), 7.32 – 7.28 (m, 3H), 7.26 – 7.22 (m, 3H), 7.12 (dd, *J* = 12.3, 6.4 Hz, 1H), 6.32 (dd, *J* = 12.4, 1.3 Hz, 1H), 4.81 – 4.72 (m, 1H), 3.28 (dd, *J* = 21.7, 3.2 Hz, 2H), 1.34 (d, *J* = 6.2 Hz, 3H), 1.26 (d, *J* = 6.3 Hz, 3H). **^{13}C NMR** (126 MHz, $CDCl_3$) δ 137.13 (d, *J* = 6.3 Hz, CH), 134.1 (C), 130.7 (d, *J* = 9.3 Hz, C), 129.9 (d, *J* = 6.7 Hz, CH), 128.7 (2 x CH), 128.6 (CH), 128.6 (CH), 127.2 (CH), 127.1 (d, *J* = 3.7 Hz, 2 x CH), 126.0 (2 x CH), 116.9 (d, *J* = 9.5 Hz, CH), 72.0 (d, *J* = 7.5 Hz, CH), 34.0 (d, *J* = 138.8 Hz, CH_2), 23.99 (d, *J* = 3.6 Hz, CH_3), 23.81 (d, *J* = 5.4 Hz, CH_3). **^{31}P NMR** (202 MHz, $CDCl_3$) δ 24.17. **HRMS** (APCI) Calc. for $C_{18}H_{22}O_3P$ [M+H$^+$]: 317.1301, found 317.1298.

(*E*)-Methyl styryl benzylphosphonate (3l)

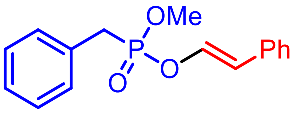

Obtained from **1l** and **2a** as a yellow oil after column chromatography following the general procedure in 31% yield.

^{1}H NMR (500 MHz, $CDCl_3$) δ 7.36 – 7.23 (m, 10H), 7.12 (dd, *J* = 12.3, 6.4 Hz, 1H), 6.34 (dd, *J* = 12.4, 1.4 Hz, 1H), 3.78 (d, *J* = 11.1 Hz, 3H), 3.32 (d, *J* = 21.8 Hz, 2H). **^{13}C NMR** (126 MHz, $CDCl_3$) δ 137.1 (d, *J* = 6.3 Hz, CH), 134.0 (C), 130.3 (d, *J* = 9.3 Hz, C) 130.0 (d, *J* = 6.8 Hz, 2 x CH), 128.8 (d, *J* = 3.2 Hz, CH), 128.7 (2 x CH), 127.3 (2 x CH), 127.4 (CH), 126.0 (2 x CH),

117.2 (d, J = 9.6 Hz, CH), 53.0 (d, J = 7.3 Hz, CH_3), 33.2 (d, J = 138.1 Hz, CH_2). **^{31}P NMR** (202 MHz, $CDCl_3$) δ 27.07. **HRMS** (APCI) Calc. for $C_{16}H_{18}O_3P$ [M+H$^+$]: 289.098, found 289.0991.

(*E*)-Ethyl 4-fluorostyryl phenylphosphonate (3m)

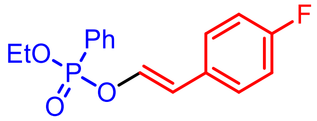

Obtained from **1d** and **2b** as a yellow oil after column chromatography following the general procedure in 75% yield.

^{1}H NMR (500 MHz, $CDCl_3$) δ 7.88 (dd, J = 13.7, 8.2, 1.3 Hz, 2H), 7.62 (tq, J = 7.4, 1.4 Hz, 1H), 7.52 (td, J = 7.8, 4.5 Hz, 2H), 7.23 (dd, J = 8.7, 5.3 Hz, 2H), 7.16 (dd, J = 12.2, 6.8 Hz, 1H), 6.99 (t, J = 8.7 Hz, 2H), 6.37 (dd, J = 13.5, 1.2 Hz, 1H), 4.32 – 4.24 (m, 2H), 1.42 (t, J = 7.1 Hz, 3H). **^{13}C NMR** (126 MHz, $CDCl_3$) δ 162.1 (d, J = 246.6 Hz, C), 136.5 (dd, J = 5.3, 2.2 Hz, 2 x CH), 133.0 (d, J = 3.1 Hz, CH), 131.8 (d, J = 10.2 Hz, 2 x CH), 130.1 (d, J = 3.4 Hz, C), 128.7 (d, J = 15.5 Hz, 2 x CH), 127.5 (d, J = 8.0 Hz, 2 x CH), 127.1 (d, J = 192.5Hz, C), 116.5 (d, J = 10.0 Hz, CH), 115.6 (d, J = 21.7 Hz, 2 x CH), 63.0 (d, J = 6.0 Hz, CH_2), 16.3 (d, J = 6.3 Hz, CH_3). **^{31}P NMR** (202 MHz, $CDCl_3$) δ 18.26. **^{19}F NMR** (282 MHz, $CDCl_3$) δ -114.9. **HRMS (APCI)** Calc. for $C_{16}H_{17}FO_3P$ [M+H$^+$]: 307.0894 found 307.0902.

(*E*)-Ethyl 4-fluorostyryl butylphosphonate (3n)

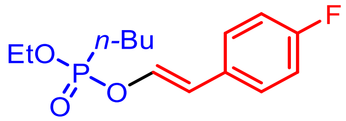

Obtained from **1a** and **2b** as a yellow oil after column chromatography following the general procedure in 65% yield.

^{1}H NMR (300 MHz, $CDCl_3$) δ 7.26 (dd, J = 8.7, 5.4 Hz, 2H), 7.18 (dd, J = 12.4, 6.6 Hz, 1H), 7.00 (t, J = 8.7 Hz, 2H), 6.33 (dd, J = 12.3, 1.4 Hz, 1H), 4.29 – 4.12 (m, 2H), 1.93 – 1.81 (m, 2H), 1.71 – 1.58 (m, 2H), 1.46 (sext, J = 7.4 Hz, 2H), 1.37 (t, J = 7.1 Hz, 3H), 0.95 (t, J = 7.4 Hz, 3H). **^{13}C NMR** (126 MHz, $CDCl_3$) δ 162.0 (d, J = 246.5 Hz, C), 136.8 (dd, J = 5.9, 2.2 Hz, 2 x CH), 130.2 (d, J = 3.3 Hz, C), 127.5 (d, J = 7.7 Hz, 2 x CH), 115.6 (d, J = 21.7 Hz, 2 x CH) 115.7 (s, CH), 62.1 (d, J = 7.0 Hz, CH_2), 25.3 (d, J = 140.3 Hz, CH_2), 24.1 (d, J = 5.3 Hz, CH_2), 23.6 (d, J = 17.6 Hz, CH_2), 16.4 (d, J = 6.1 Hz, CH_3), 13.5 (CH_3). **^{31}P NMR** (202 MHz, $CDCl_3$) δ 32.73. **^{19}F NMR** (282 MHz, $CDCl_3$) δ -115.4. **HRMS (APCI)** Calc. for $C_{14}H_{21}FO_3P$ [M+H$^+$]: 287.1112 found 287.1115.

(*E*)-Ethyl 4-methylstyryl butylphosphonate (3o)

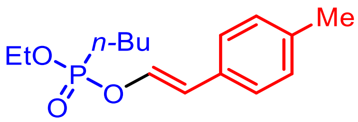

Obtained from **1a** and **2c** as a yellow oil after column chromatography following the general procedure in 94% yield.

^{1}H NMR (300 MHz, $CDCl_3$) δ 7.26 – 7.16 (m, 3H), 7.11 (d, J = 7.8 Hz, 2H), 6.34 (dd, J = 12.5, 2.1 Hz, 1H), 4.30 – 4.09 (m, 2H), 2.34 (s, 3H), 1.95 – 1.81 (m, 2H), 1.72 – 1.59 (m, 2H), 1.46 – 1.39 (m, 2H), 1.37 (t, J = 6.9 Hz, 3H), 0.94 (t, J = 7.2 Hz, 3H). **^{13}C NMR** (126 MHz, $CDCl_3$) δ 136.0 (C), 135.3 (d, J = 6.0 Hz, CH), 130.1 (C), 128.4 (2 x CH), 124.9 (2 x CH), 115.7 (d, J = 9.5 Hz, CH), 61.0 (d, J = 7.2 Hz, CH_2), 24.3 (d, J = 140.3 Hz, CH_2), 23.1 (d, J = 5.4 Hz, CH_2), 22.6 (d, J = 17.4 Hz, CH), 20.1 (CH_3), 15.4 (d, J = 6.1 Hz, CH_3), 12.5 (CH_3). **^{31}P NMR** (202 MHz, $CDCl_3$) δ 31.56. **HRMS (APCI)** Calc. for $C_{15}H_{23}O_3P$ [M+H$^+$]: 283.1458 found 283.1454.

(*E*)-Ethyl 4-(trifluoromethyl)styryl butylphosphonate (3p)

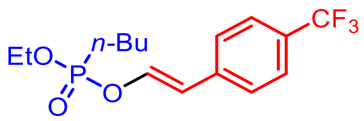

Obtained from **1a** and **2d** as a yellow oil after column chromatography following the general procedure in 70% yield.

^{1}H NMR (500 MHz, $CDCl_3$) δ 7.54 (d, *J* = 8.2 Hz, 2H), 7.38 (d, *J* = 8.5 Hz, 2H), 7.35 (dd, *J* = 12.4, 6.9 Hz, 1H), 6.36 (d, *J* = 12.3 Hz, 1H), 4.26 – 4.14 (m, 2H), 1.92 – 1.84 (m, 2H), 1.69 – 1.62 (m, 2H), 1.45 (sext, *J* = 7.3 Hz, 2H), 1.37 (t, *J* = 7.1 Hz, 3H), 0.94 (t, *J* = 7.3 Hz, 3H). **^{13}C NMR** (126 MHz, $CDCl_3$) δ 139.3 (d, *J* = 5.8 Hz, C), 138.2 (d, *J* = 1.28 Hz, CH), 129.3 (q, *J* = 32.5 Hz, C), 126.3 (2 x CH), 125.9 (q, *J* = 3.8 Hz, 2 x CH), 124.4 (q, *J* = 271.9 Hz, C), 115.5 (d, *J* = 9.6 Hz, CH), 62.4 (d, *J* = 7.1 Hz, CH_2), 26.6 (d, *J* = 138 Hz, CH_2), 24.4 (d, *J* = 5.4 Hz, CH_2), 23.9 (d, *J* = 16.08 Hz, CH_2), 16.6 (d, *J* = 12.5 Hz, CH_3), 13.8 (d, J = 1.2 Hz, CH_3). **^{31}P NMR** (202 MHz, $CDCl_3$) δ 31.95. **^{19}F NMR** (282 MHz, $CDCl_3$) δ -62.66. **HRMS (APCI)** Calc. for $C_{15}H_{21}F_3O_3P$ [M+H$^+$]: 337.1181 found 337.1176.

6. ^{1}H NMR and ^{13}C NMR spectra

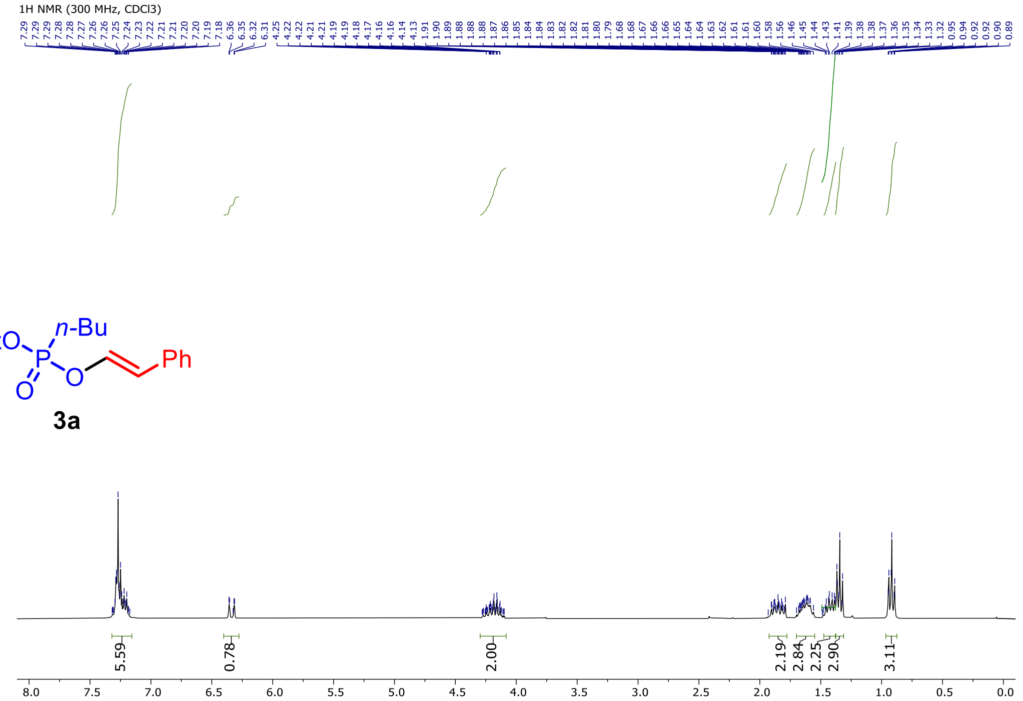

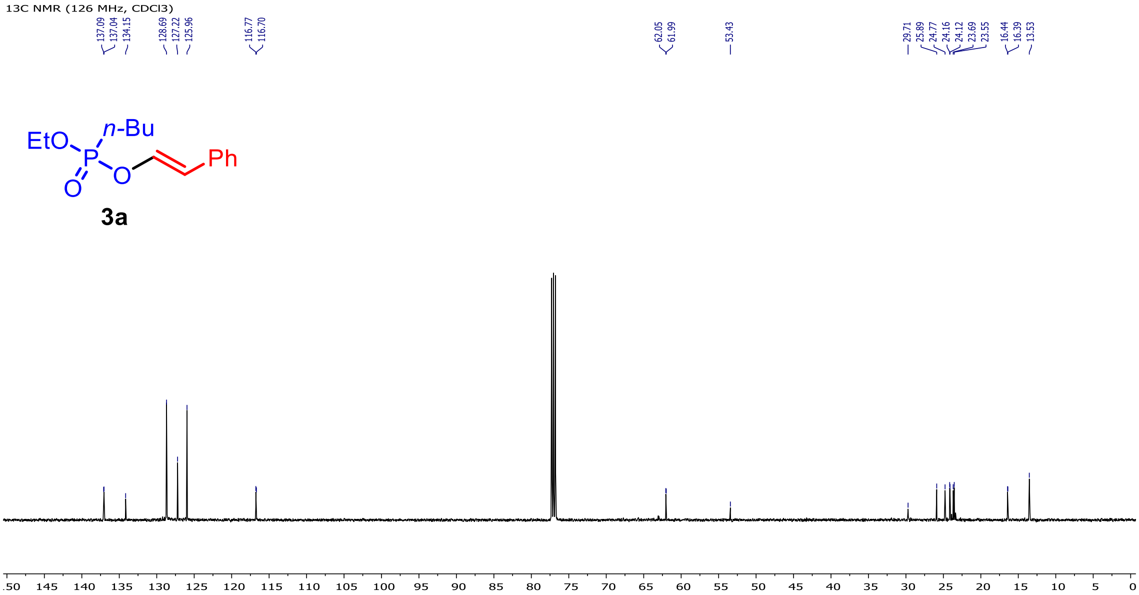

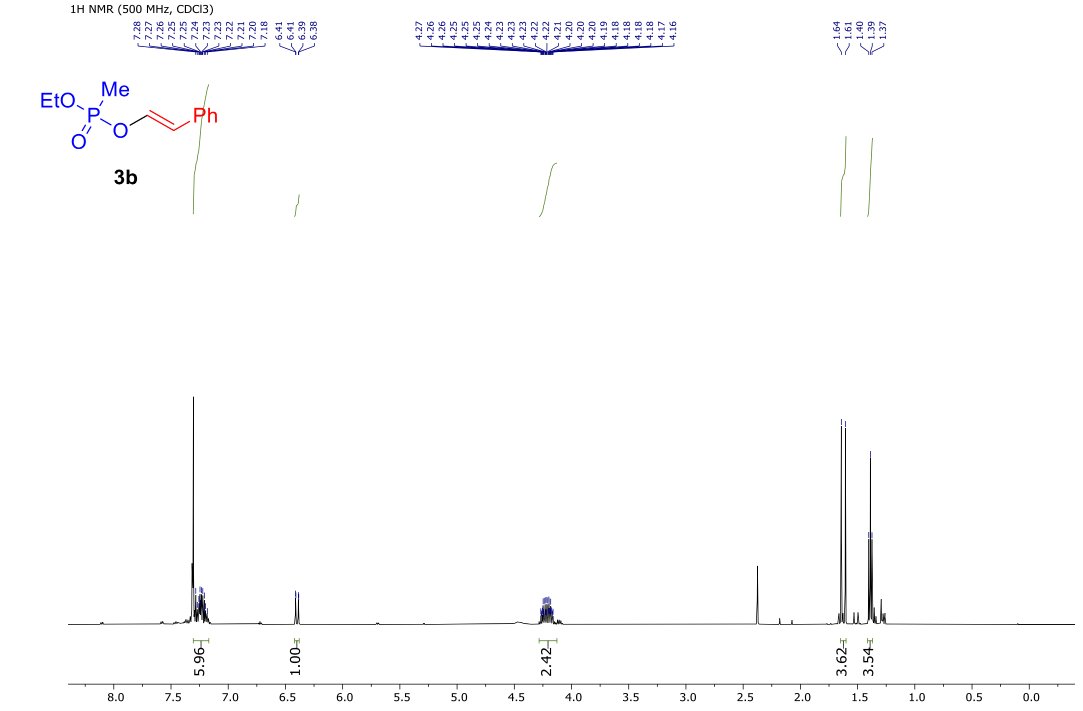
1H NMR (500 MHz, CDCl3)
7.28 7.27 7.26 7.25 7.25 7.25 7.24 7.23 7.23 7.23 7.22 7.21 7.20 7.18
6.41 6.41 6.39 6.38
4.27 4.26 4.26 4.25 4.25 4.25 4.24 4.23 4.23 4.23 4.22 4.22 4.21 4.20 4.20 4.20 4.19 4.18 4.18 4.18 4.18 4.17 4.16
1.64 1.61 1.40 1.39 1.37
EtO
Me
P
O
O
Ph
3b
5.96
1.00
2.42
3.62
3.54
8.0 7.5 7.0 6.5 6.0 5.5 5.0 4.5 4.0 3.5 3.0 2.5 2.0 1.5 1.0 0.5 0.0

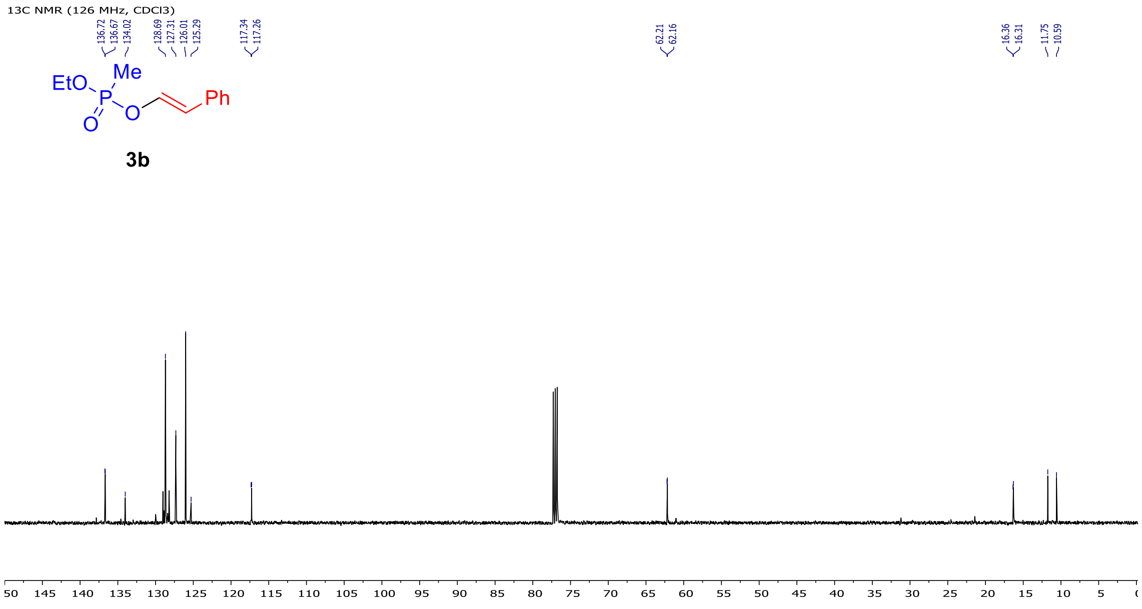
13C NMR (126 MHz, CDCl3)
136.72 136.67 134.02
128.69 127.31 126.01 125.29
117.34 117.26
62.21 62.16
16.36 16.31 11.75 10.59
EtO
Me
P
O
O
Ph
3b
150 145 140 135 130 125 120 115 110 105 100 95 90 85 80 75 70 65 60 55 50 45 40 35 30 25 20 15 10 5 0

1H NMR (300 MHz, CDCl3)

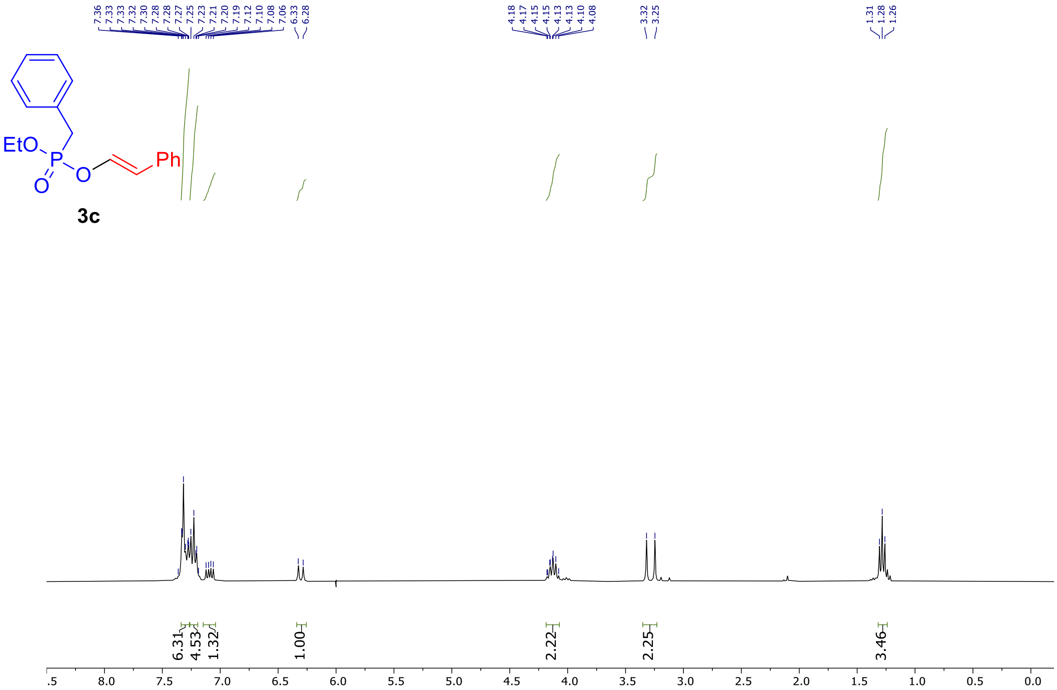

13C NMR (126 MHz, CDCl3)

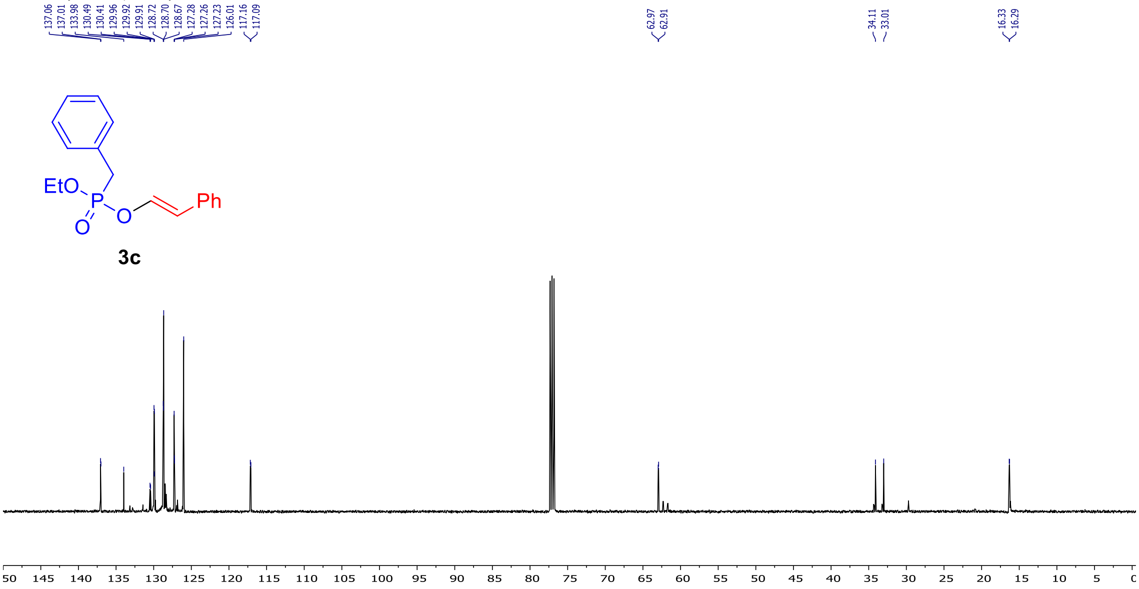

1H NMR (500 MHz,CDCl3)

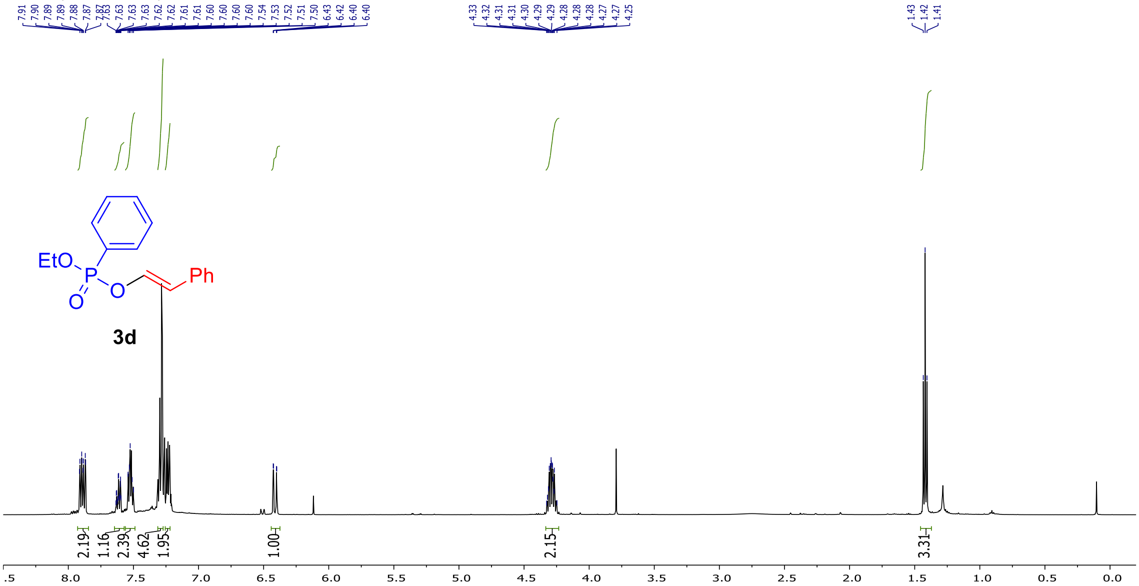

13C NMR (126 MHz, CDCl3)

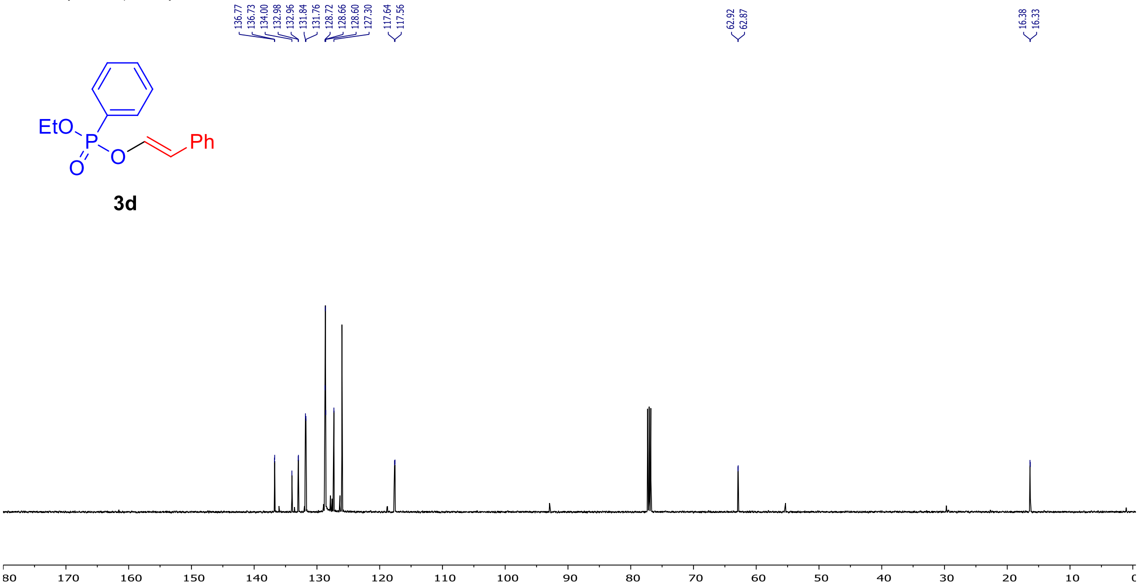

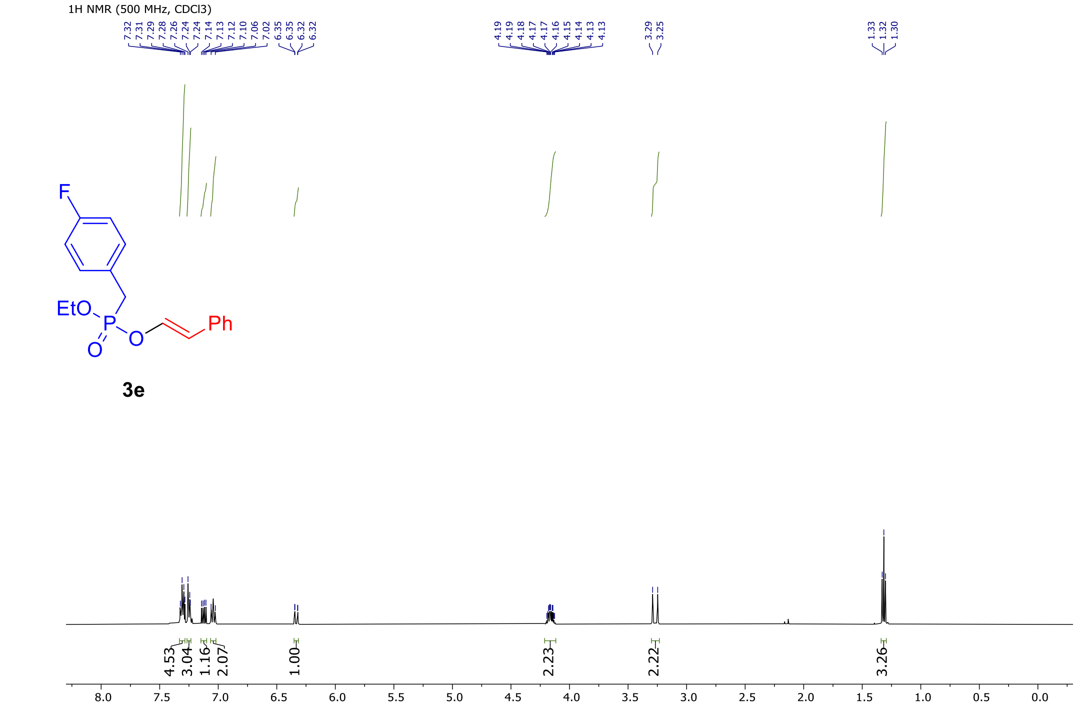
1H NMR (500 MHz, CDCl3)
7.32 7.31 7.29 7.28 7.26 7.24 7.24 7.14 7.13 7.12 7.10 7.06 7.02 6.35 6.35 6.32 6.32
4.19 4.19 4.18 4.17 4.17 4.16 4.15 4.14 4.13 4.13
3.29 3.25
1.33 1.32 1.30
F
EtO
P
O
O
Ph
3e
4.53 3.04 1.16 2.07 1.00 2.23 2.22 3.26

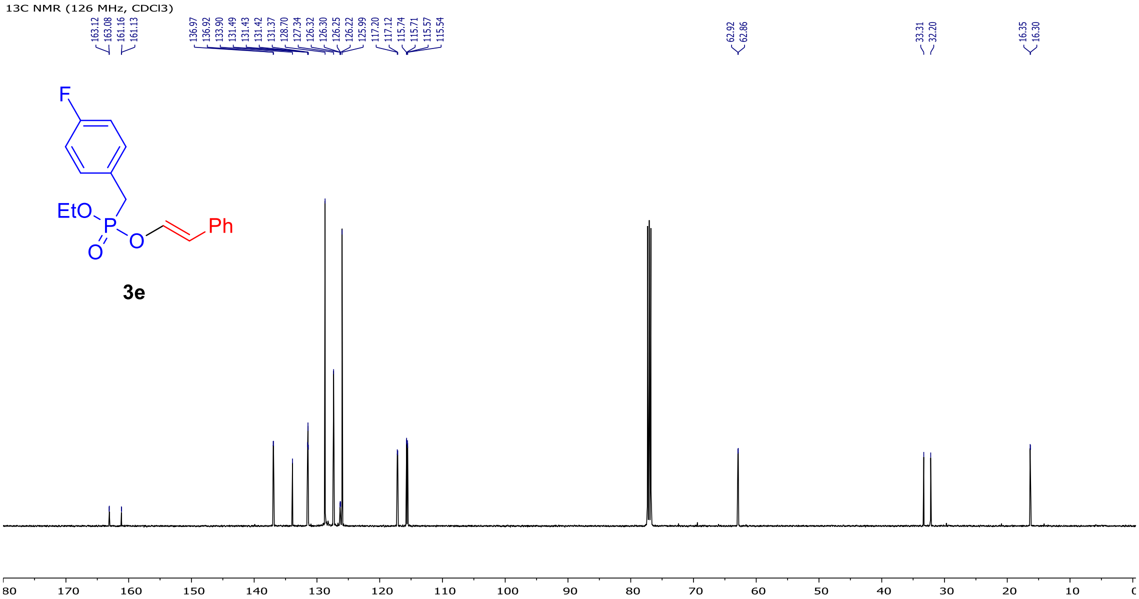
13C NMR (126 MHz, CDCl3)
163.12 163.08 161.16 161.13
136.97 136.92 133.90 131.49 131.43 131.42 131.37 128.70 127.34 126.32 126.30 126.25 126.22 125.99 117.20 117.12 115.74 115.71 115.57 115.54
62.92 62.86
33.31 32.20
16.35 16.30
F
EtO
P
O
O
Ph
3e

1H NMR (500 MHz, CDCl3)

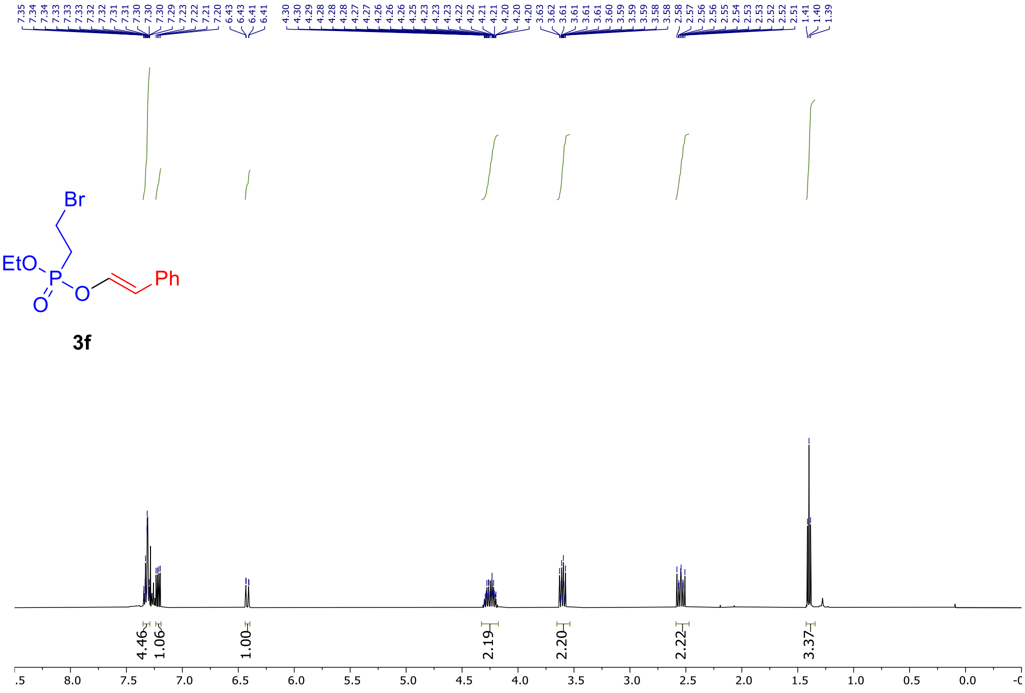

13C NMR (126 MHz, CDCl3)

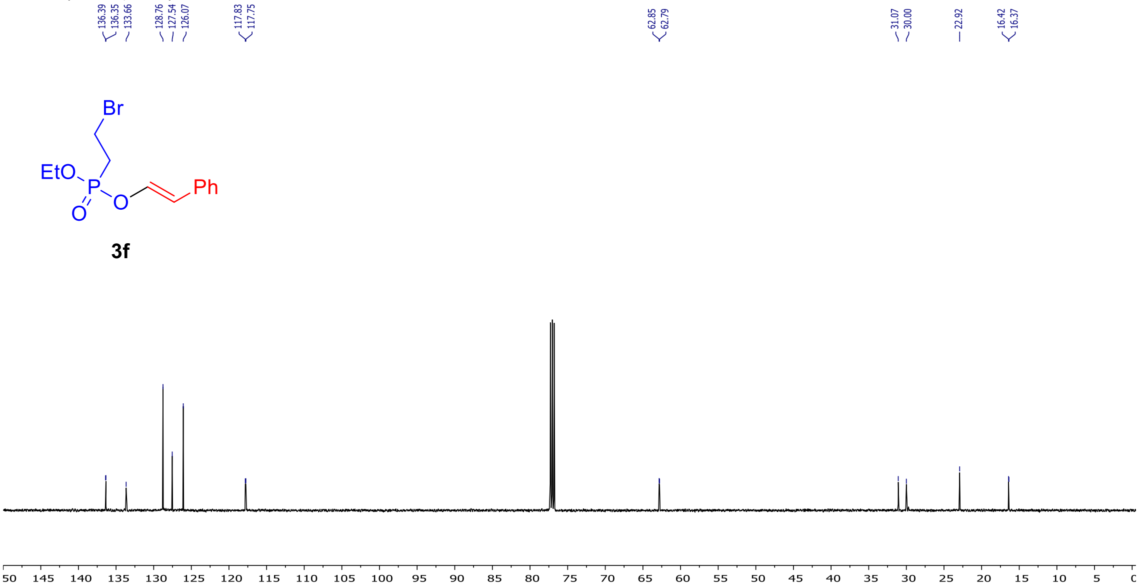

1H NMR (500 MHz, CDCl3)

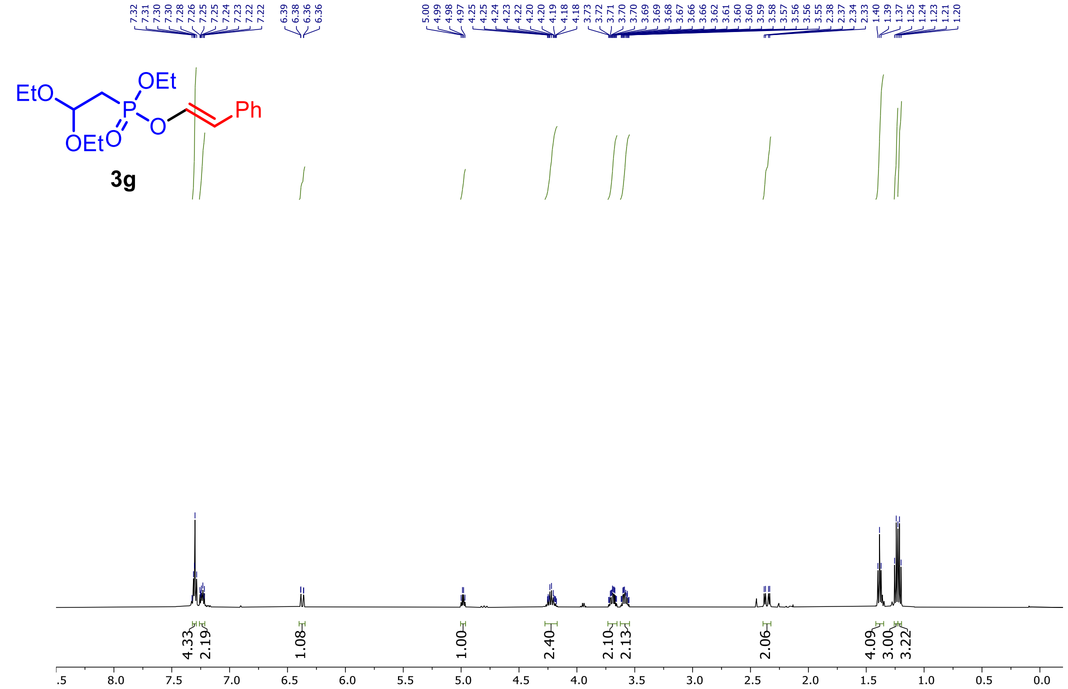

13C NMR (75 MHz, CDCl3)

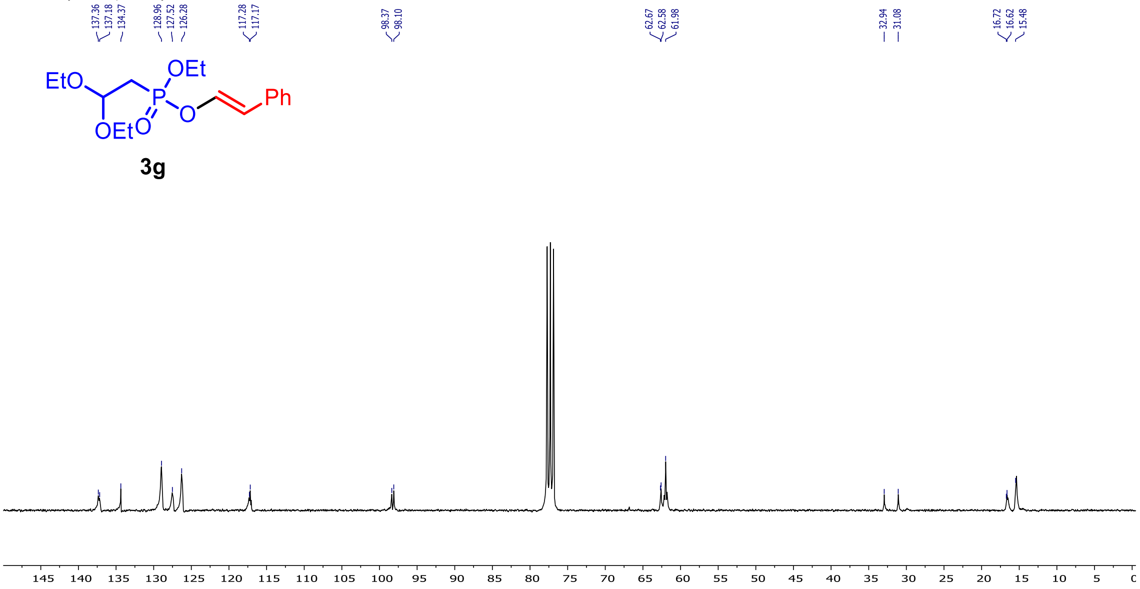

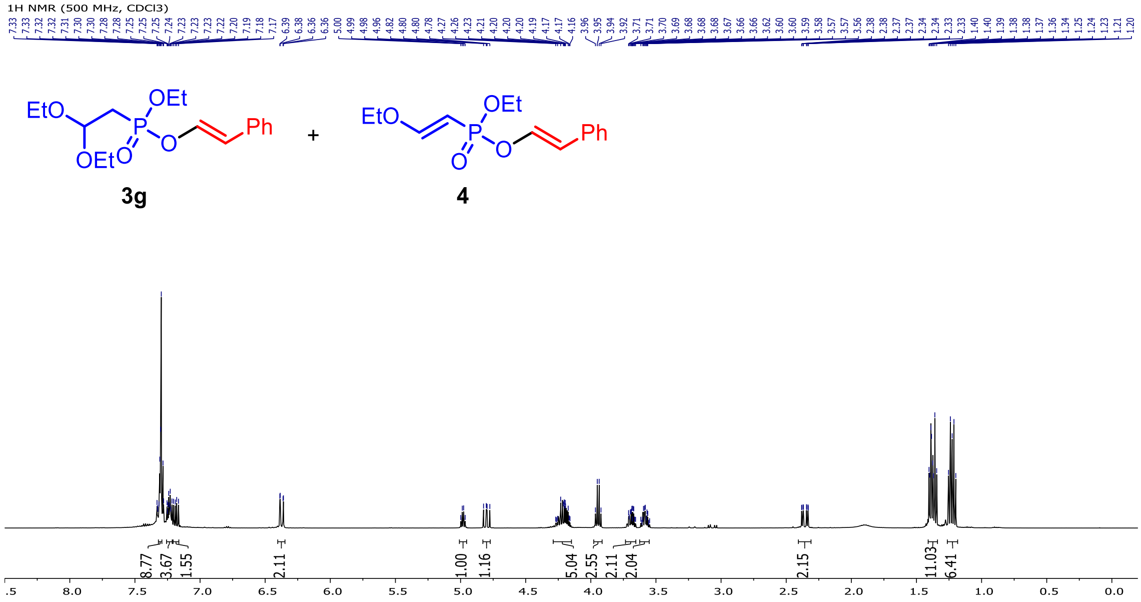
1H NMR (500 MHz, CDCl3)
EtO
OEt
OEt
Ph
3g
4
8.77
3.67
1.55
2.11
1.00
1.16
5.04
2.55
2.11
2.04
2.15
11.03
6.41

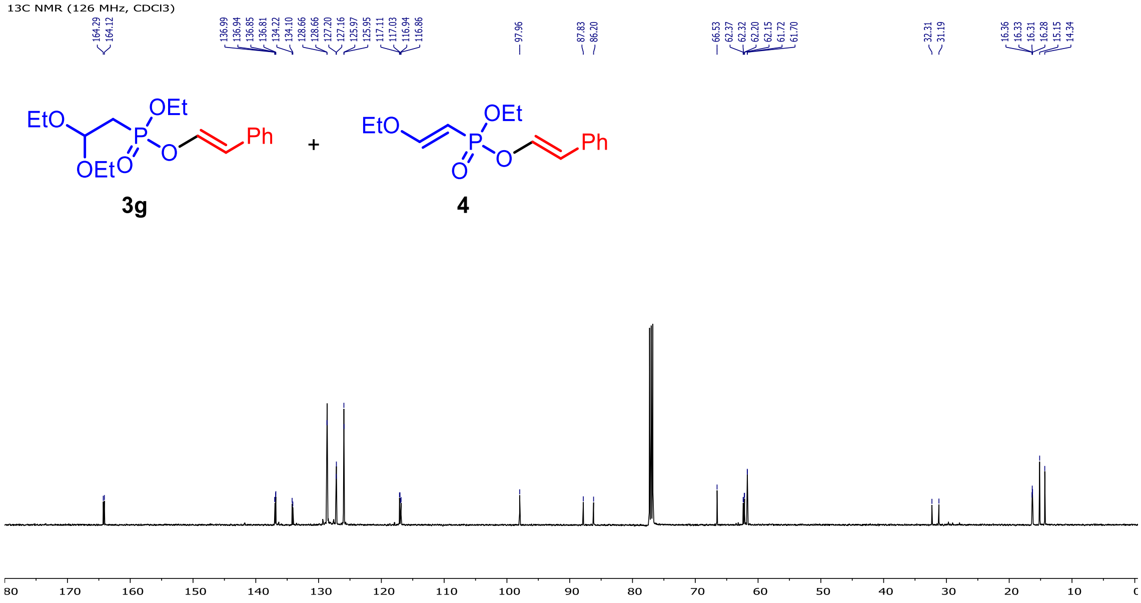
13C NMR (126 MHz, CDCl3)
EtO
OEt
OEt
Ph
3g
4

1H NMR (300 MHz, CDCl3)

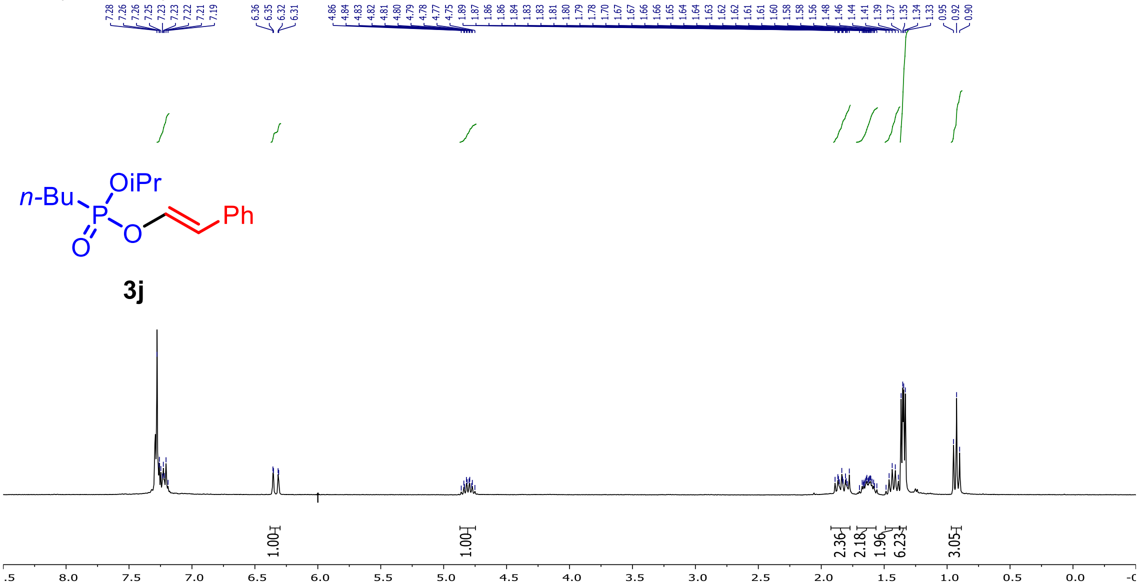

13C NMR (126 MHz, CDCl3)

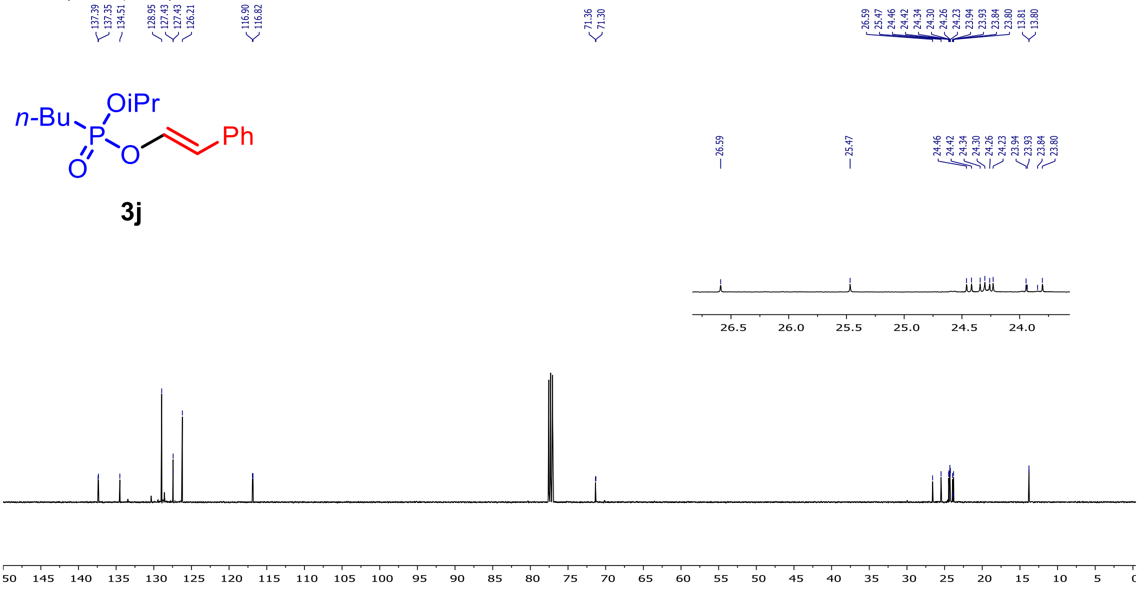

1H NMR (500 MHz, CDCl3)

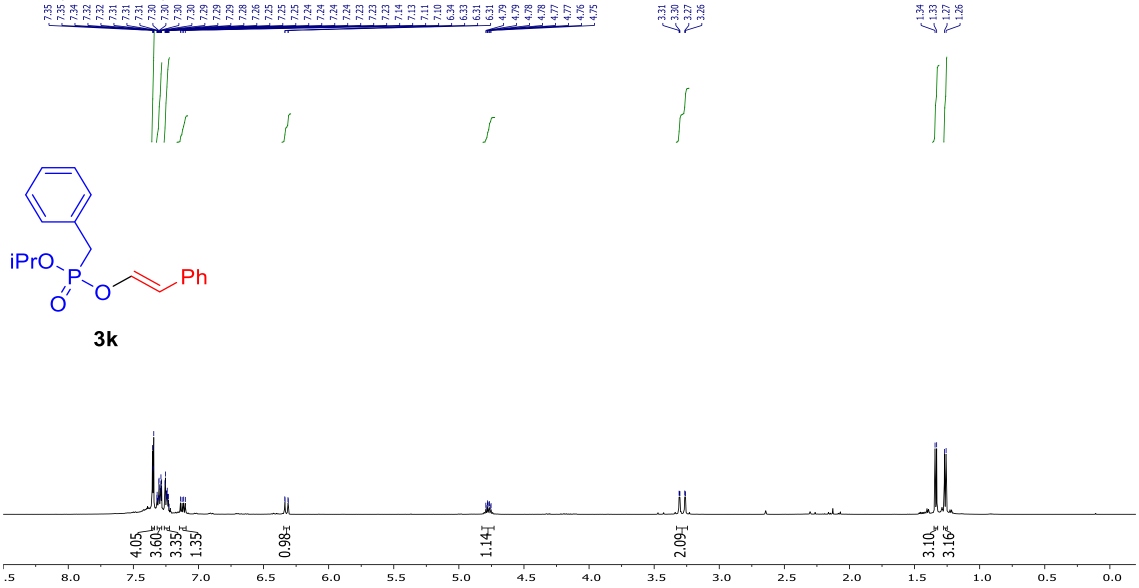

13C NMR (126 MHz, CDCl3)

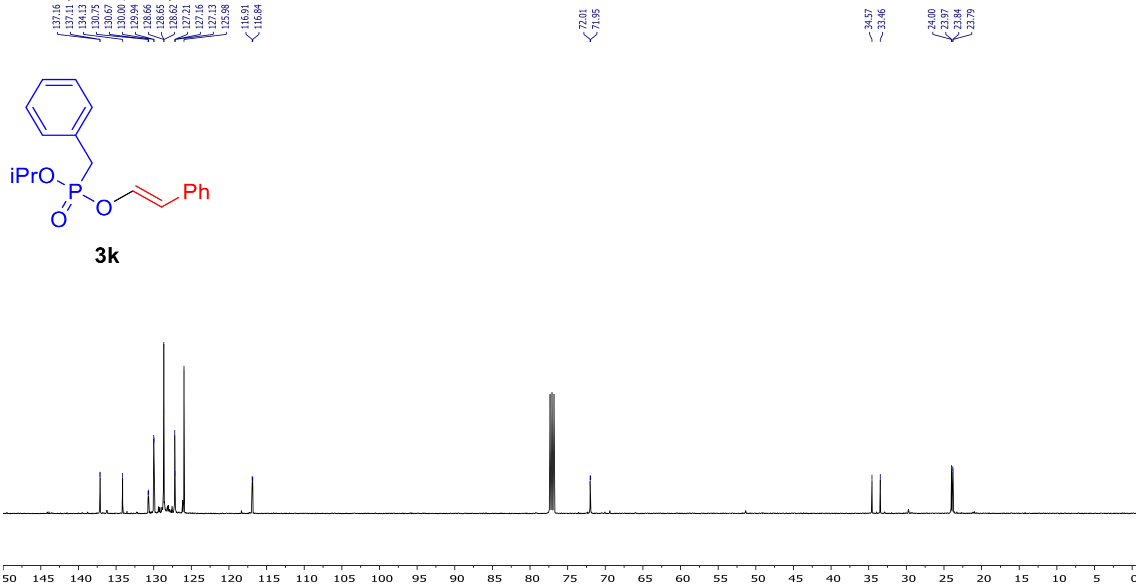

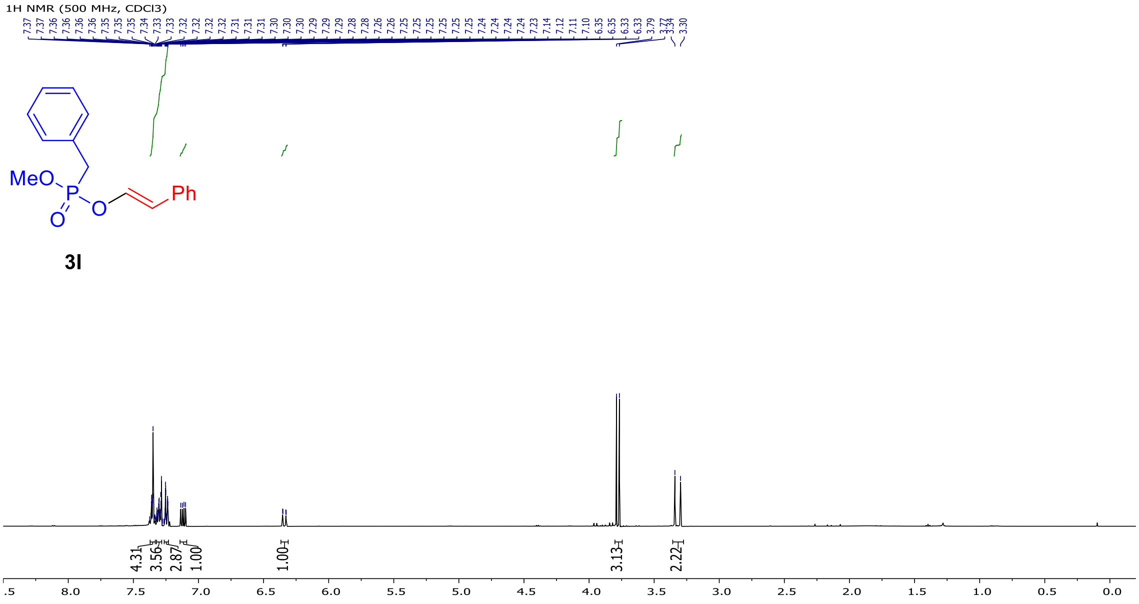
1H NMR (500 MHz, CDCl3)
MeO
P
O
O
Ph
3l
4.31
3.56
2.87
1.00
1.00
3.13
2.22

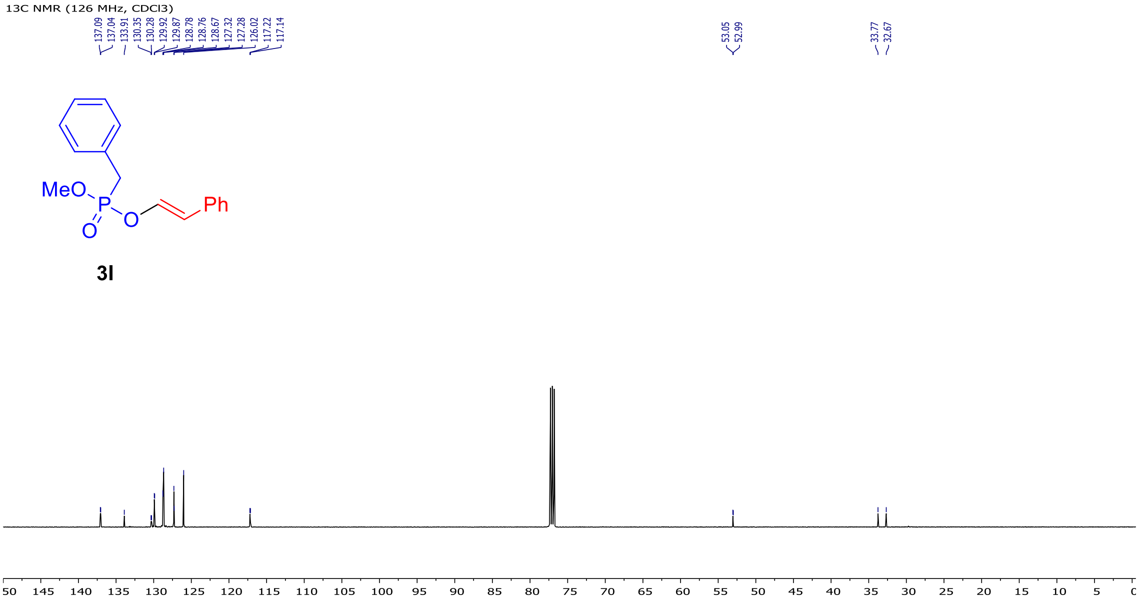
13C NMR (126 MHz, CDCl3)
MeO
P
O
O
Ph
3l

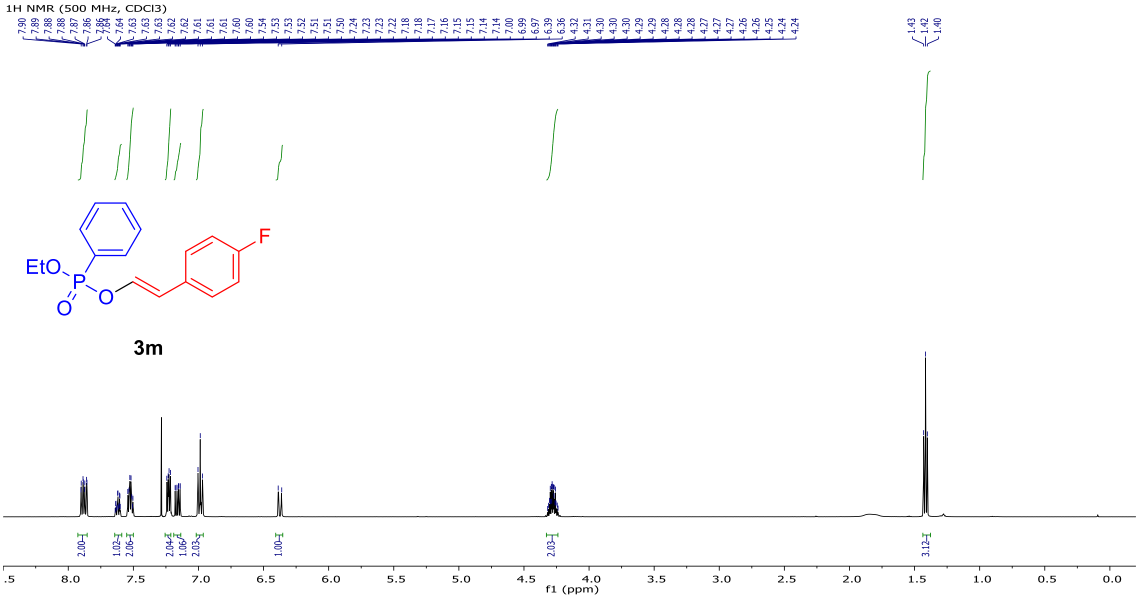
1H NMR (500 MHz, CDCl3)
7.90 7.89 7.88 7.88 7.87 7.86 7.86 7.64 7.64 7.63 7.63 7.63 7.62 7.61 7.61 7.61 7.60 7.60 7.54 7.53 7.53 7.52 7.51 7.51 7.50 7.24 7.23 7.23 7.22 7.18 7.17 7.16 7.15 7.15 7.14 7.14 7.00 6.99 6.97 6.39 6.36 4.32 4.31 4.30 4.30 4.29 4.29 4.28 4.28 4.28 4.27 4.27 4.26 4.26 4.25 4.24 4.24
1.43 1.42 1.40
EtO
P
O
O
F
3m
2.00 1.02 2.06 2.04 1.06 2.03 1.00 2.03 3.12
f1 (ppm)

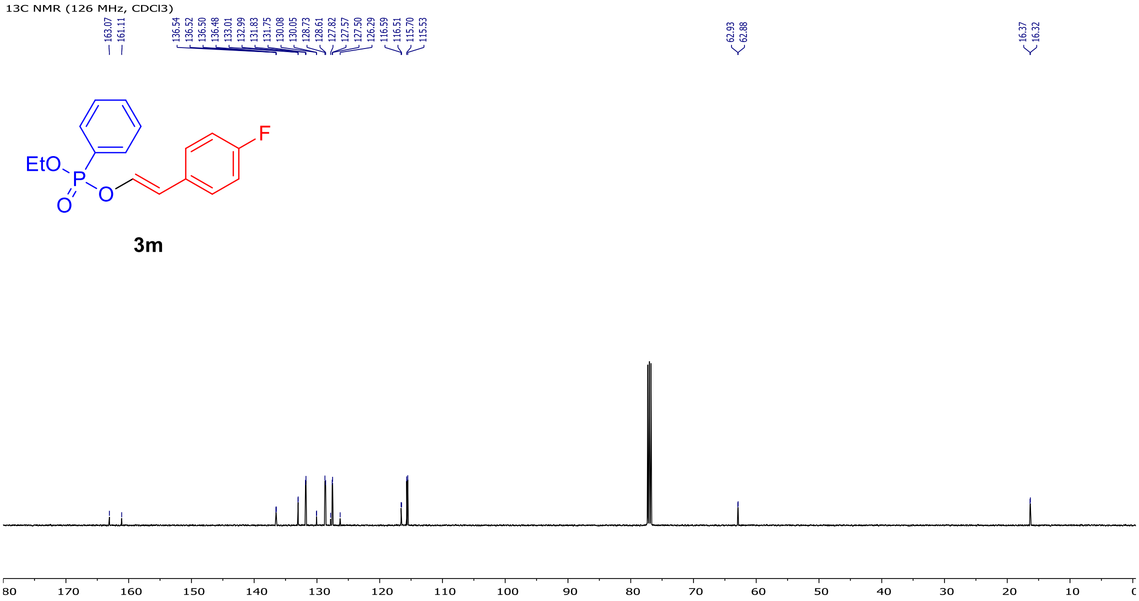
13C NMR (126 MHz, CDCl3)
163.07 161.11
136.54 136.52 136.50 136.48 133.01 132.99 131.83 131.75 130.08 130.05 128.73 128.61 127.82 127.57 127.50 126.29 116.59 116.51 115.70 115.53
62.93 62.88
16.37 16.32
EtO
P
O
O
F
3m

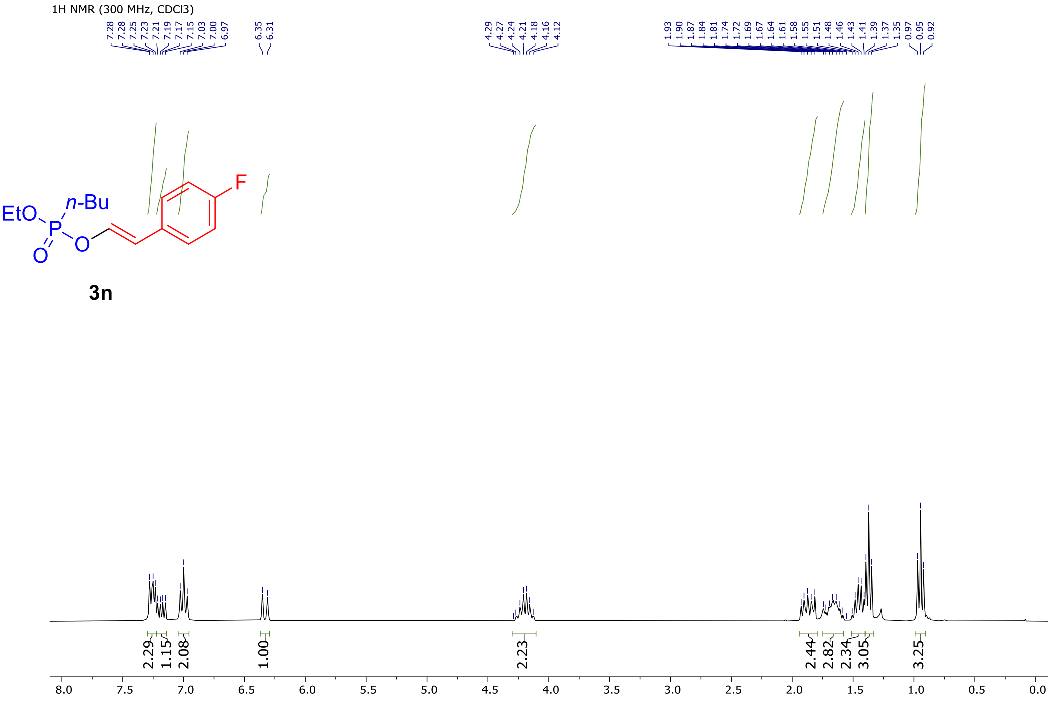
1H NMR (300 MHz, CDCl3)
EtO
n-Bu
F
3n

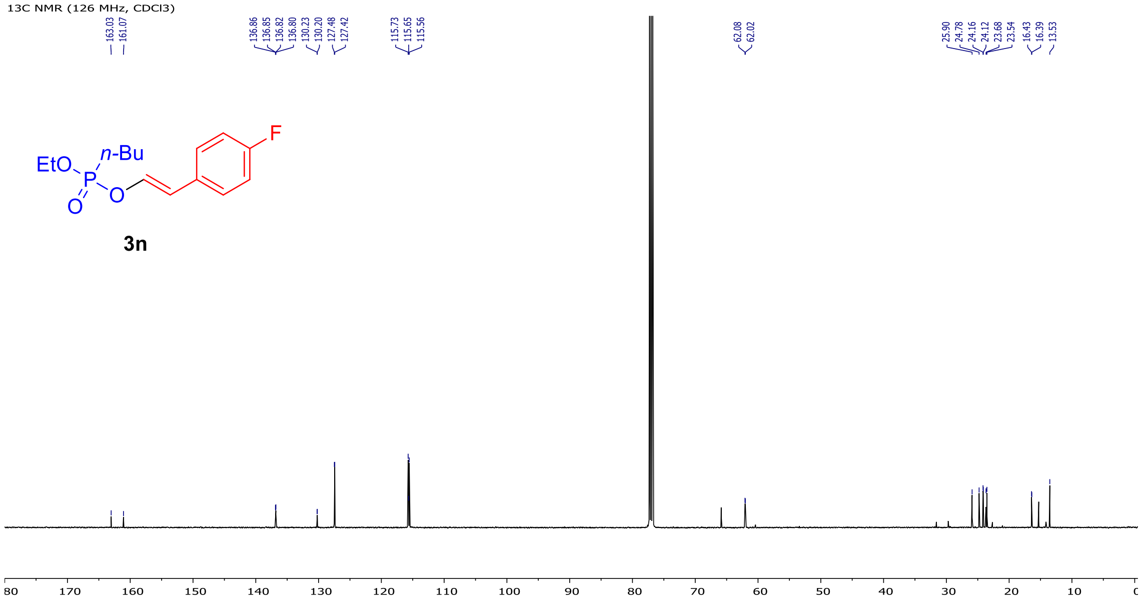
13C NMR (126 MHz, CDCl3)
EtO
n-Bu
F
3n

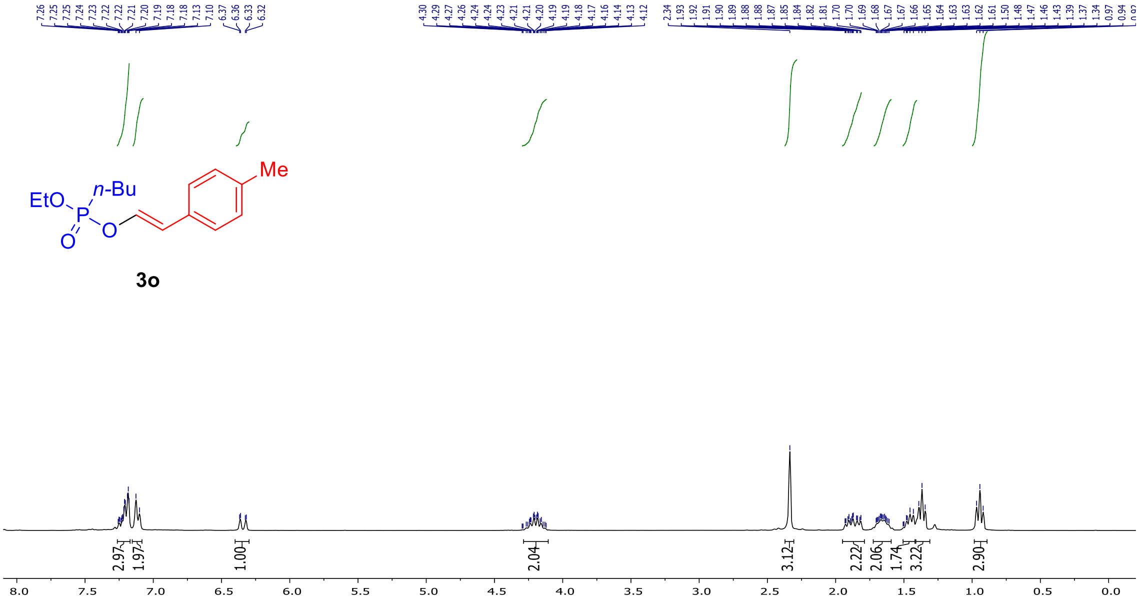
EtO
n-Bu
P
O
O
Me
3o

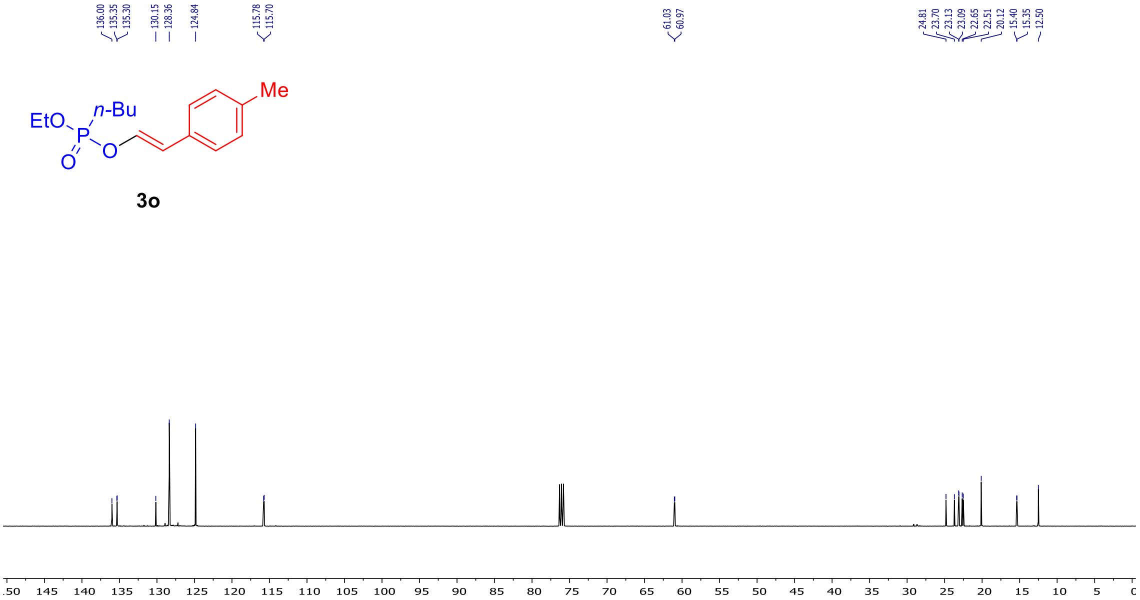
EtO
n-Bu
P
O
O
Me
3o

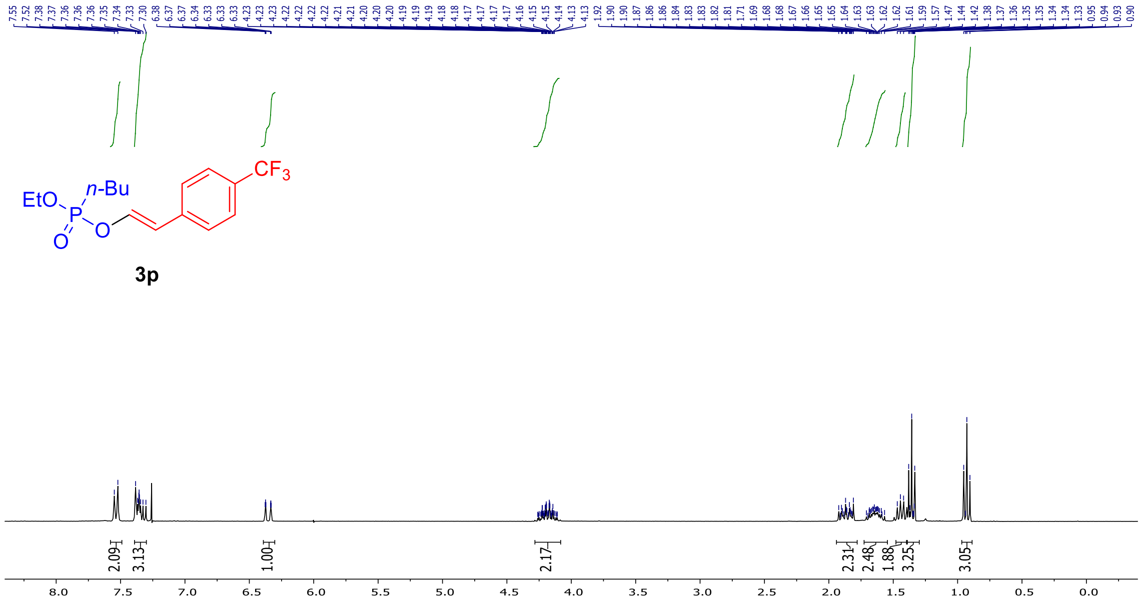
EtO
n-Bu
P
O
O
CF3
3p

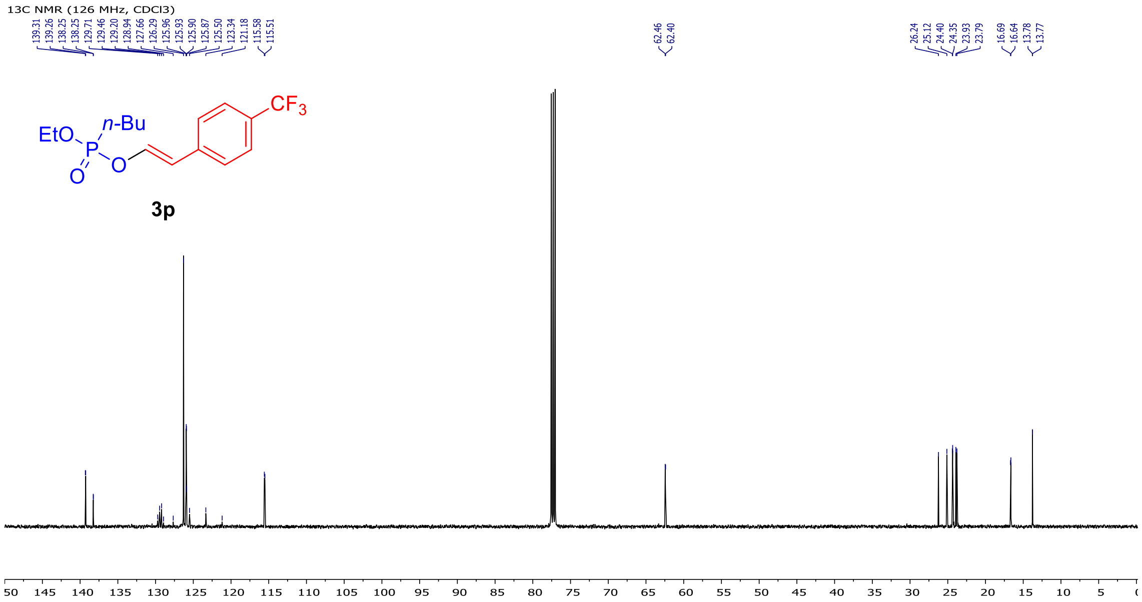
13C NMR (126 MHz, CDCl3)
EtO
n-Bu
P
O
O
CF3
3p
